# Lateral Gene Transfer Drives Metabolic Flexibility in the Anaerobic Methane-Oxidizing Archaeal Family *Methanoperedenaceae*

**DOI:** 10.1128/mBio.01325-20

**Published:** 2020-06-30

**Authors:** Andy O. Leu, Simon J. McIlroy, Jun Ye, Donovan H. Parks, Victoria J. Orphan, Gene W. Tyson

**Affiliations:** a Australian Centre for Ecogenomics, School of Chemistry and Molecular Biosciences, University of Queensland, St. Lucia, Australia; b Centre for Microbiome Research, School of Biomedical Sciences, Queensland University of Technology (QUT), Translational Research Institute, Woolloongabba, Australia; c Department of Geological and Planetary Sciences, California Institute of Technology, Pasadena, California, USA; CEH, Oxford University

**Keywords:** ANME, AOM, comparative genomics, methane, *Methanoperedenaceae*

## Abstract

Anaerobic oxidation of methane (AOM) is an important biological process responsible for controlling the flux of methane into the atmosphere. Members of the archaeal family *Methanoperedenaceae* (formerly ANME-2d) have been demonstrated to couple AOM to the reduction of nitrate, iron, and manganese. Here, comparative genomic analysis of 16 *Methanoperedenaceae* metagenome-assembled genomes (MAGs), recovered from diverse environments, revealed novel respiratory strategies acquired through lateral gene transfer (LGT) events from diverse archaea and bacteria. Comprehensive phylogenetic analyses suggests that LGT has allowed members of the *Methanoperedenaceae* to acquire genes for the oxidation of hydrogen and formate and the reduction of arsenate, selenate, and elemental sulfur. Numerous membrane-bound multiheme *c*-type cytochrome complexes also appear to have been laterally acquired, which may be involved in the direct transfer of electrons to metal oxides, humic substances, and syntrophic partners.

## Abstract (precis)

AOM by microorganisms limits the atmospheric release of the potent greenhouse gas methane and has consequent importance for the global carbon cycle and climate change modeling. While the oxidation of methane coupled to sulfate by consortia of anaerobic methanotrophic (ANME) archaea and bacteria is well documented, several other potential electron acceptors have also been reported to support AOM. In this study, we identify a number of novel respiratory strategies that appear to have been laterally acquired by members of the *Methanoperedenaceae*, as they are absent from related archaea and other ANME lineages. Expanding the known metabolic potential for members of the *Methanoperedenaceae* provides important insight into their ecology and suggests their role in linking methane oxidation to several global biogeochemical cycles.

## INTRODUCTION

Anaerobic oxidation of methane (AOM) is an important microbiological process moderating the release of methane from anoxic waters and sediments into the atmosphere (1–4). Several diverse uncultured microbial lineages have been demonstrated to facilitate AOM. The bacterium “*Candidatus* Methylomirabilis oxyfera” is proposed to couple AOM to denitrification from nitrite, generating oxygen from nitric oxide for the activation of methane (5). Different lineages of anaerobic methanotrophic (ANME) archaea are hypothesized to mediate AOM through the reversal of the methanogenesis pathway and conserve energy using mechanisms similar to those found in methylotrophic and aceticlastic methanogens (6). Unlike methanogens, most of these ANMEs encode a large repertoire of multiheme *c*-type cytochromes (MHCs), which are proposed to mediate direct interspecies electron transfer to syntrophic sulfate-reducing bacteria (SRB) (7, 8) and/or the reduction of metal oxides and humic acids (9–12).

Currently, several clades within the archaeal phylum *Euryarchaeota* have been shown to be capable of anaerobic methanotrophy and include ANME-1a and -1b, ANME-2a to -2c, *Methanoperedenaceae* (formerly known as ANME-2d), and ANME-3 (13–15). Marine ANME lineages are often observed to form consortia with SRB, with ANME-1 and ANME-2 (a, b, and c) being associated with multiple genera within the *Desulfobacterales* and *Desulfobulbaceae* (13, 16–20), thermophilic ANME-1 being associated with “*Candidatus* Desulfofervidus auxilii” (8, 21), and ANME-3 being associated with SRBs of the *Desulfobulbus* (22). While members of the family *Methanoperedenaceae* have also recently been associated with SRB of the family *Desulfobulbaceae* in a freshwater lake sediment (23), they also appear to oxidize methane independently using a range of electron acceptors. The type species of this family, “*Candidatus* Methanoperedens nitroreducens,” was originally enriched in a bioreactor and shown to couple AOM to the reduction of nitrate via a laterally transferred nitrate reductase (15). Subsequently, “*Candidatus* Methanoperedens sp.” strain BLZ1 was also found to encode a laterally transferred nitrite reductase, which is also present in the genome of “*Ca.* Methanoperedens nitroreducens,” potentially allowing these microorganisms to couple AOM to dissimilatory nitrate reduction to ammonia (DNRA) (24). More recently, three novel species belonging to the *Methanoperedenaceae* were enriched in bioreactors demonstrated to couple AOM to the reduction of insoluble iron or manganese oxides (9, 12). These microorganisms did not encode dissimilatory nitrate reduction pathways but instead were inferred to use multiple unique MHCs during metal-dependent AOM to facilitate the transfer of electrons to the metal oxides (9, 12), consistent with the extracellular electron transfer mechanisms proposed for marine ANME organisms (7, 8). Bioreactor performance and 16S rRNA gene amplicon data have also been used to suggest that members of the *Methanoperedenaceae* are capable of AOM coupled to the reduction of selenate and chromium(VI), although this remains to be confirmed with more direct evidence (25, 26). Notably, members of the *Methanoperedenaceae* have been observed to facilitate AOM coupled to multiple terminal electron acceptors within the same natural sediment (27). Individual members of the family can possess such metabolic flexibility, with a lab-enriched species shown to couple AOM to the reduction of nitrate, iron, and manganese oxides (10). Given the relatively poor genomic representation of the *Methanoperedenaceae* and the lack of detailed physiological studies of its members, it is likely that considerable metabolic diversity for the lineage remains to be discovered.

In this study, comparative analysis was conducted on 16 *Methanoperedenaceae* metagenome-assembled genomes (MAGs) recovered from various environments to investigate the metabolic diversity and versatility of the family and to understand the evolutionary mechanisms responsible for these adaptations. These analyses indicate that members of the *Methanoperedenaceae* have acquired a large number of genes through lateral gene transfer (LGT) that potentially allow AOM to be coupled to a wide range of electron acceptors, suggesting that their role in methane oxidation extends beyond environments with nitrate and metal oxides.

## RESULTS AND DISCUSSION

### Expanding the genomic representation of the *Methanoperedenaceae*.

In order to explore the metabolic diversity within the *Methanoperedenaceae*, comparative genomic analysis was performed on both publicly available and newly acquired MAGs (Table 1). The publicly available genomes include six MAGs recovered from bioreactors where AOM is coupled to the reduction of nitrate (“*Ca.* Methanoperedens nitroreducens”; M.Nitro [15], BLZ2 [28], and IPS-1 [29]), iron (“*Ca.* Methanoperedens ferrireducens”; M.Ferri [9]), and manganese (“*Ca.* Methanoperedens manganicus” and “*Ca.* Methanoperedens manganireducens,” Mn-1 and Mn-2, respectively [12]). Also included are two environmental MAGs recovered from groundwater samples from the Horonobe and Mizunami underground research laboratories in Japan (HGW-1 and MGW-1) (30, 31). In order to recover additional genomes belonging to the family, GraftM (32) was used to screen public metagenome sequence data sets from the NCBI for *Methanoperedenaceae*-related 16S rRNA and *mcrA* gene sequences. Subsequent assembly and genome binning on data sets found to contain *Methanoperedenaceae*-like sequences led to the recovery of an additional eight MAGs belonging to the family. Six of these were from arsenic-contaminated groundwater samples (ASW-1-6), and a further two were from sediment and groundwater samples from a copper mine tailings dam (CMD-1 and CMD-2). All 16 MAGs are highly complete (≥87.4%), with low contamination (≤5.9%) based on 228 *Euryarchaeota*-specific marker genes (Table 1) (33). These genomes vary in GC content from 40.2 to 50.7% and range in size from 1.45 to 3.74 Mbp.

**TABLE 1:** Characteristics of the metagenome-assembled genomes

Bin ID	Genome size (mbp)	No. of scaffolds	*N*_50_ (scaffolds; bp)	Strain hetero- geneity^*a*^	Compl. (%)^*a*^	Cont. (%)^*a*^	%GC	No. of CDSs^*e*^	Source environment and associated publication	Accession no.^*b*^	16S rRNA gene?
ASW-1	1.52	271	7,386	0.0	87.5	0.0	47.8	1,946	Arsenic-contaminated groundwater, Bangladesh (104)	SRR1563167, SRR1564103, SRR1573565, SRR1573578, SAMN10961276	N
ASW-2	2.63	157	28,058	25.0	94.4	4.8	48.0	2,944	Arsenic-contaminated groundwater, Bangladesh (104)	SRR1563167, SRR1564103, SRR1573565, SRR1573578, SAMN10961277	N
ASW-3	2.51	100	44,967	0.0	100.0	1.3	50.7	2,892	Arsenic-contaminated groundwater, Bangladesh (104)	SRR1563167, SRR1564103, SRR1573565, SRR1573578, SAMN10961278	N
ASW-4	2.24	155	24,336	0.0	97.1	0.7	43.2	2,464	Arsenic-contaminated groundwater, Bangladesh (104)	SRR1563167, SRR1564103, SRR1573565, SRR1573578, SAMN10961279	N
ASW-5	2.97	221	19,046	0.0	95.0	2.6	48.9	3,353	Arsenic contaminated groundwater, Bangladesh (104)	SRR1563167, SRR1564103, SRR1573565, SRR1573578, SAMN10961280	N
ASW-6	2.19	68	56,691	66.7	99.4	2.0	46.6	2,472	Arsenic-contaminated groundwater, Bangladesh (104)	SRR1563167, SRR1564103, SRR1573565, SRR1573578, SAMN10961281	Y
BLZ1^*c*^	3.74	514	17,508	13.33	96.73	6.56	40.2	4,659	AOM-nitrate bioreactor, Netherlands (24)	LKCM00000000.1	Y
BLZ2	3.74	85	74,304	0.0	99.4	4.6	40.3	4,041	AOM-nitrate reactor, Netherlands (28)	GCA_002487355.1	N
CMD-1	1.85	116	27,949	100.0	98.0	0.7	44.9	2,261	Copper mine tailings dam, Brazil (105)	SRR5161805, SRR5161795, SAMN10961282	N
CMD-2	1.45	221	9,704	0.0	88.4	0.0	44.1	1,786	Copper mine tailings dam, Brazil (105)	SRR5161805, SRR5161795, SAMN10961283	N
HGW-1	2.00	128	24,496	33.3	96.4	2.0	43.2	2,288	Groundwater samples, Japan (31)	GCA_002839545.1	Y
IPS-1	3.52	250	27,331	10.0	97.7	5.9	44.1	3,970	AOM-nitrate bioreactor seeded from paddy field soil, Italy (29)	GCA_900196725.1	Y
M.Ferri	2.91	59	88,069	0.0	98.7	1.3	40.8	3,019	AOM-iron bioreactor, Australia (9)	GCA_003104905.1	Y
M.Nitro	3.20	10	54,4976	0.0	99.7	1.3	43.2	3,428	AOM-nitrate bioreactor, Australia (15)	GCA_000685155.1	Y
MGW-1	2.08	161	17,186	0.0	97.4	3.6	44.8	2,488	Groundwater samples, Japan (30)	Not available^*d*^	N
Mn-1	3.59	68	87,551	0.0	100.0	1.3	40.6	3,737	AOM-manganese bioreactor, Australia (12)	SAMN10872768	N
Mn-2	3.32	116	49,809	0.0	99.4	4.6	42.9	3,684	AOM-manganese bioreactor, Australia (12)	SAMN10872769	N

a Completeness (compl.), contamination (cont.), and strain heterogeneity were estimated using CheckM (33).

b Genome accession numbers. For the MAGs assembled in this study the SRA accession numbers are also given.

c The BLZ1 genome was not used in analyses, as it is almost identical to the BLZ2 genome (99.5% ANI) and has inferior completeness and contamination values. The BLZ1 bioreactor was the parent system of the BLZ2 bioreactor.

d This genome was provided by Yohey Suzuki and is associated with the study of Ino and colleagues (30).

e CDSs, coding sequences; N, no; Y, yes.

**FIG 1:**
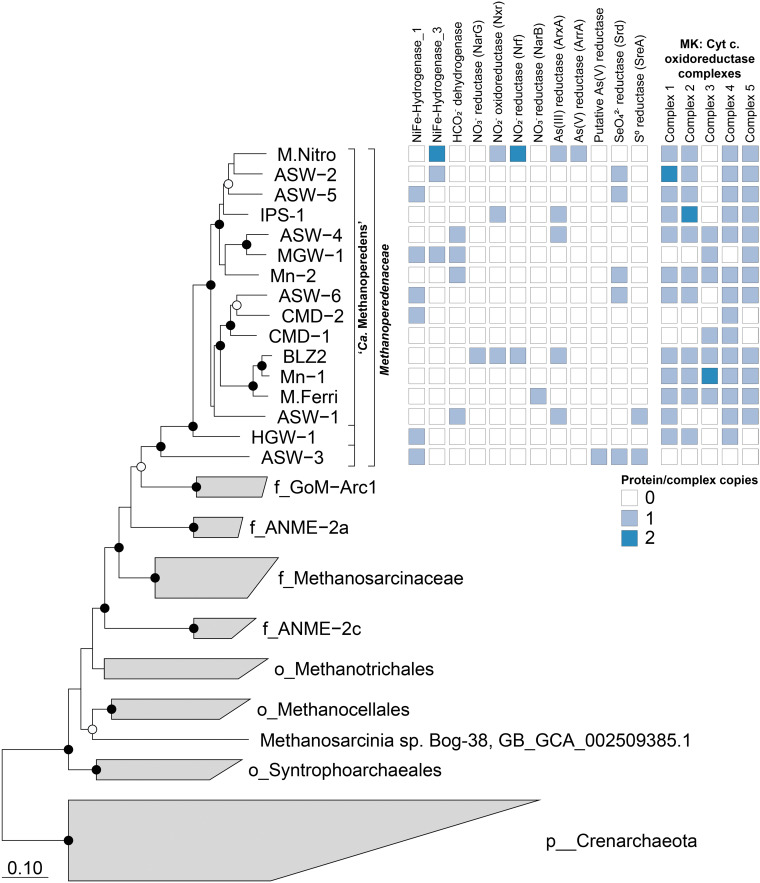
Phylogenetic placement of the *Methanoperedenaceae* MAGs and distribution of potential terminal electron acceptors. The genome tree was inferred using maximum likelihood with a concatenated set of 122 archaeon-specific marker genes. Black and white dots indicate >90% and >70% bootstrap values, respectively. The scale bar represents amino acid nucleotide changes. Based on GTDB-Tk, the family *Methanoperedenaceae* includes three genera, including “*Ca.* Methanoperedens,” which are denoted with brackets. The table to the right of the tree shows the presence/absence of genes associated with potential terminal electron acceptors in each corresponding *Methanoperedenaceae* genome.

A genome tree including 1,199 publicly available archaeal genomes, based on a concatenated set of 122 marker genes (34), confirmed the phylogenetic placement of the 16 MAGs within the *Methanoperedenaceae*. The genome tree supports that these MAGs form a monophyletic clade sister to the GoM-Arc1 genomes (Fig. 1). These genomes likely represent three separate genera within the family, based on their placement within a reference tree, relative evolutionary distance, FastANI distance, and average amino acid identity (AAI [35]; 61.3 to 89.2%) (see Fig. S1 in the supplemental material). All MAGs were classified as members of the genus “*Ca*. Methanoperedens,” except HGW-1 and ASW-3, which appear to represent independent genus-level lineages (Fig. 1). Phylogenetic analysis of the six MAGs containing 16S rRNA genes was consistent with the genome tree (Fig. S2), supporting their classification as members of the *Methanoperedenaceae* family.

10.1128/mBio.01325-20.1 FIG S1 Average amino acid identity (AAI%) for the *Methanoperedenaceae* genomes. AAI was calculated between each pair of genomes using CompareM. Download FIG S1, PDF file, 0.2 MB. Copyright © 2020 Leu et al. 2020 Leu et al. This content is distributed under the terms of the Creative Commons Attribution 4.0 International license.

10.1128/mBio.01325-20.2 FIG S2 16S rRNA gene-based phylogenetic placement of the *Methanoperedenaceae* MAGs. The 16S rRNA genes extracted from the *Methanoperedenaceae* MAGs from this study are indicated in red. Support values calculated via nonparametric bootstrapping. The scale bar represents numbers of changes per nucleotide position. Download FIG S2, PDF file, 0.5 MB. Copyright © 2020 Leu et al. 2020 Leu et al. This content is distributed under the terms of the Creative Commons Attribution 4.0 International license.

### Potential electron donors used by the *Methanoperedenaceae*.

**FIG 2:**
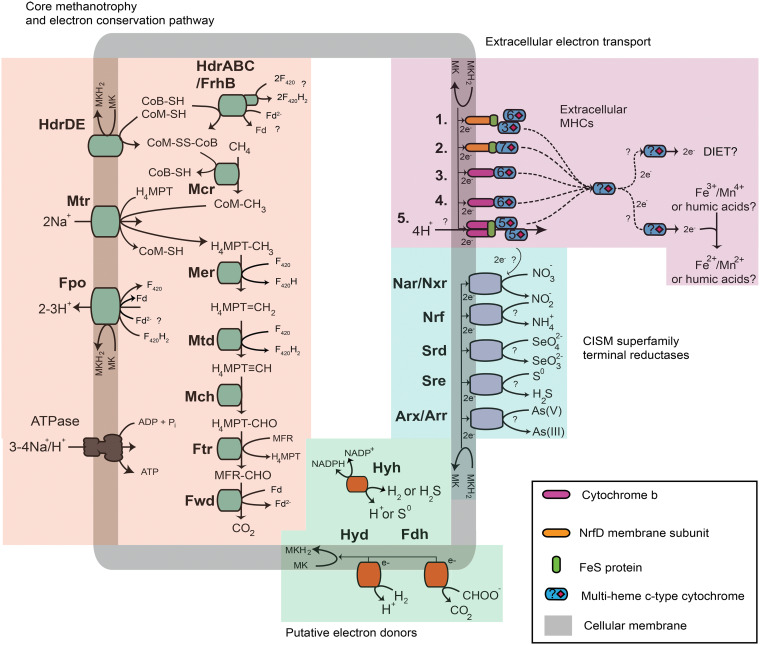
Metabolic capabilities of the *Methanoperedenaceae*. Key metabolic pathways for the anaerobic oxidation of methane, energy conservation mechanisms, hydrogen and formate oxidation, and electron acceptors found within the pangenome of the *Methanoperedenaceae*. Numbers 1 to 5 indicate the different menaquinone:cytochrome *c* oxidoreductases conserved in the *Methanoperedenaceae* MAGs (Data Set S1A). Abbreviations for enzymes and cofactors in the figure are as follows: H_4_MPT, tetrahydromethanopterin; MFR, methanofuran; Fwd, formyl-methanofuran dehydrogenase; Ftr, formylmethanofuran/H_4_MPT formyltransferase; Mch, methenyl-H_4_MPT cyclohydrolase; Mtd, F_420_-dependent methylene H4MPT dehydrogenase; Mer, F_420_-dependent methylene-H_4_MPT reductase; Mtr, Na^+^-translocating methyl-H_4_MPT:coenzyme M (CoM) methyltransferase; Mcr, methyl-CoM reductase; F_420_, F_420_ coenzyme; Fd, ferredoxin; CoM-SH, coenzyme M; CoB-HS, coenzyme B; Hdr, heterodisulfide reductase; Fpo, F_420_H_2_ dehydrogenase; Hyd, type 1 NiFe hydrogenase; Hyh, type 3b NiFe hydrogenase; Fdh, formate dehydrogenase; Nar, nitrate reductase; Nrf, nitrite reductase; Srd, selenate reductase; Sre, sulfur reductase; Arx, arsenite oxidase; Arr, arsenate reductase; DIET, direct interspecies electron transfer.

**FIG 3:**
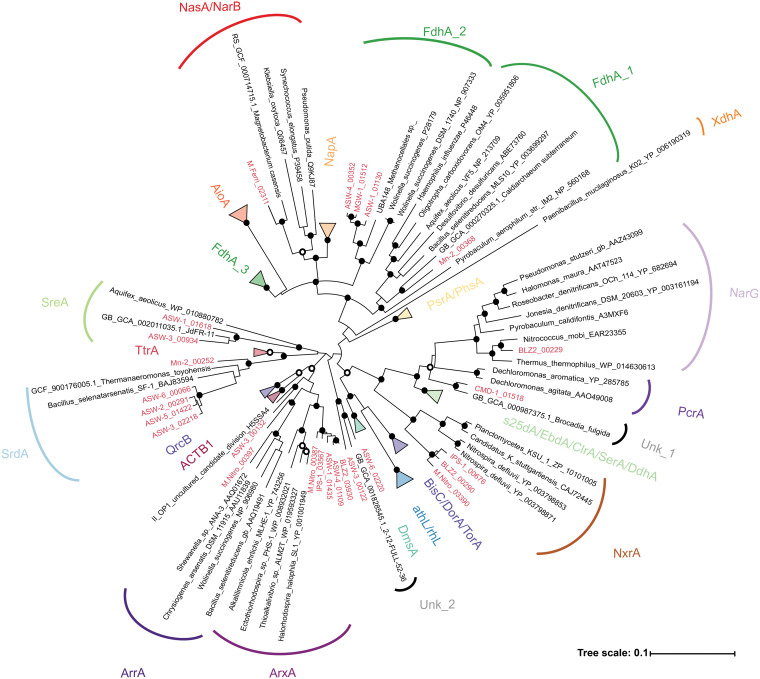
Phylogenetic analysis of the catalytic subunits of the CISM superfamily. Putative genes recovered from the *Methanoperedenaceae* are indicated in red. The gene tree was inferred by maximum likelihood, and support values were calculated via nonparametric bootstrapping. Black and white dots indicate >90% and >70% bootstrap support, respectively. The scale bar represents amino acid changes. ACTB1, alternate complex III, domain of subunit B; ArrA, arsenate reductase; ArxA, arsenite oxidase; AthL, pyrogallol hydroxytransferase; BisC, biotin sulfoxide reductase; ClrA, chlorate reductase; EbdA, ethylbenzene dehydrogenase; s25dA, C25 dehydrogenase; DmsA, DMSO reductase; DorA, DMSO reductase; NapA, nitrate reductase; NarG, nitrate reductase; NasA, assimilatory nitrate reductase; NarB, assimilatory nitrate reductase; NxrA, nitrite oxidoreductase; PsrA, polysulfide reductase; PhsA, thiosulfate reductase; QrcB, quinone reductase complex; TtrA tetrathionate reductase; DmsA, PcrA, perchlorate reductase; SrdA, Selenate reductase; SreA, sulfur reductase; TorA, trimethylamine *N*-oxide (TMAO) reductase; XdhA, xanthine dehydrogenase; FdhA, formate dehydrogenase; rhL, resorcinol hydroxylase; Unk, unknown putative reductase. Amino acid sequences are included in Data Set S1B.

Metabolic reconstruction of the *Methanoperedenaceae* MAGs showed that all genomes encoded the central methanogenesis pathway, inclusive of the methyl coenzyme M (methyl-CoM) reductase, supporting their potential for the complete oxidation of methane to CO_2_ (Fig. 2 and Fig. S3). The annotation of membrane-bound formate dehydrogenases (FdhAB) in four of the *Methanoperedenaceae* MAGs (Mn-2, ASW-4, ASW-1, and MGW-1) (Fig. 3) suggests that some members of the family may also oxidize formate (*E*_0_ [CO_2_/HCOO^−^] = −430 mV) (36). As the enzyme is reversible, these species may also potentially produce formate as a supplementary electron sink during AOM, as suggested for Mn-2 (12). Formate was proposed as a putative electron shuttle between ANME-1 organisms and their syntrophic partner SRB, based on the annotation and expression of *fdhAB* in ANME-1, but this has not been supported with physiological studies (37, 38). The putative formate dehydrogenase encoded in the Mn-2 MAG is phylogenetically related to an FdhA found in the genome of *Caldiarchaeum subterraneum*, while those encoded by ASW-4, ASW-1, and MGW-1 appear to be more similar to FdhA of *Methanocellaceae* archaeon UBA148 (Fig. 3).

10.1128/mBio.01325-20.3 FIG S3 Phylogenetic analysis of methyl-coenzyme reductase subunit A (McrA). Putative genes recovered from the *Methanoperedenaceae* are indicated in red. The gene tree was inferred using maximum likelihood, and support values were calculated via nonparametric bootstrapping. The scale bar represents amino acid changes. Download FIG S3, PDF file, 0.1 MB. Copyright © 2020 Leu et al. 2020 Leu et al. This content is distributed under the terms of the Creative Commons Attribution 4.0 International license.

10.1128/mBio.01325-20.10 DATA SET S1 Sequences, identifiers, and statistics for genes used in the comparative analyses of the *Methanoperedenaceae* MAGs. (A) Genes encoding proteins involved in the methane oxidation pathway, energy conservation, and other metabolic pathways as shown in Fig. 2. (B) Amino acid sequences used in the CISM superfamily gene tree (Fig. 3). Amino acid sequences include curated sequences from Swiss-Prot and the work of Castelle et al. (C. J. Castelle, L. A. Hug, K. C. Wrighton, B. C. Thomas, et al., Nat Commun 4:2120, 2013, https://doi.org/10.1038/ncomms3120) and closely related sequences from GTDB r83 protein reference database (D. H. Parks, M. Chuvochina, D. W. Waite, C. Rinke, et al., Nat Biotechnol 36:996–1004, 2018, https://doi.org/10.1038/nbt.4229). (C) Amino acid sequences used in the catalytic subunits of the energy-converting NiFe hydrogenase. Amino acid sequences include curated sequences from Greening et al. (C. Greening, A. Biswas, C. R. Carere, C. J. Jackson, et al., ISME J 10:761–777, 2016, https://doi.org/10.1038/ismej.2015.153) and closely related sequences from the GTDB r83 protein reference database. (D) Genes encoding putative NiFe hydrogenase maturation proteins. (E) Best blastp hits of Fpo dehydrogenase subunits to the IMG database. Blastp hits show that divergent Fpo subunits are present in the *Methanoperedenaceae* MAGs, as seen in Fig. S6. Top blast hits to “*Ca.* Methanoperedens”-like protein sequences were excluded. (F) General statistic of multiheme *c*-type cytochromes (MHCs) in the ANME genomes. (G) MHC general statistics for all bacterial and archaeal families in the GTDB v89 database. Download Data Set S1, XLSX file, 0.2 MB. Copyright © 2020 Leu et al. 2020 Leu et al. This content is distributed under the terms of the Creative Commons Attribution 4.0 International license.

The use of hydrogen (H_2_; *E*_0_ = –414 mV [39]) as an electron source was previously suggested for MGW-1 and HGW-1, which encode group 1 membrane-bound NiFe hydrogenase complexes, composed of a NiFe catalytic subunit, a FeS electron transfer subunit, and a membrane-bound *b*-type cytochrome (30, 31). These hydrogenases, along with similar group 1 NiFe hydrogenases identified in the ASW-6 and CMD-2 MAGs, form a monophyletic clade with those encoded by the MAG for “*Candidatus* Hydrothermarchaeota” (JdFR-18), which belongs to the archaeal phylum *Hydrothermarchaeota* (40), and several members of the *Halobacterota* (Fig. S4A). The ASW-3 and ASW-5 MAGs encode group 1 NiFe hydrogenases that are basal to Vho/Vht/Vhx hydrogenases encoded by members of the genus *Methanosarcina* (41). As the ASW-5 NiFe hydrogenase does not encode a *b*-type cytochrome (Fig. S4B), it is unclear how electrons are derived from hydrogen. In addition to the membrane-bound NiFe hydrogenases, the M.Nitro MAG was found to carry genes for two different sets of group 3b cytoplasmic hydrogenases (Fig. S4A). The MGW-1 (30) and ASW-2 MAGs also encode group 3b hydrogenases, which have been implicated in hydrogen evolution and NADP (NADPH) reduction (42). Similar complexes have also been shown to have hydrogen oxidation and elemental-sulfur-reducing capabilities (42–44). It is unknown how these group 3b hydrogenases contribute to energy conservation given their predicted cytoplasmic localization. The functionality of the annotated group 1 and 3 NiFe hydrogenases is supported by the identification of the NiFe binding motifs (L1 and L2) on their NiFe catalytic subunits and the annotation of all or most of the hydrogenase maturation genes (*hypA* to *-F*) on the same *Methanoperedenaceae* MAGs (Data Set S1D). The potential for some *Methanoperedenaceae* to couple the oxidation of hydrogen and/or formate to the reduction of exogenous electron acceptors would be advantageous with the dynamic availability of methane in natural environments (45).

10.1128/mBio.01325-20.4 FIG S4 Phylogenetic analysis of the subunits of the NiFe hydrogenases annotated in the *Methanoperedenaceae* genomes. (A) Analysis of the catalytic subunits of the energy-converting NiFe hydrogenases. (B) Analysis of the *b*-type cytochrome in the group 1 NiFe hydrogenases. Putative genes recovered from the *Methanoperedenaceae* are indicated in red. The gene trees were inferred using maximum likelihood and support values calculated via nonparametric bootstrapping. The reference sequences of group 1 and group 3 NiFe hydrogenases were acquired from the work of Greening et al. (C. Greening, A. Biswas, C. R. Carere, C. J. Jackson, et al., ISME J 10:761–777, 2016, https://doi.org/10.1038/ismej.2015.153) and the GTDB v83 reference sequences (D. H. Parks, M. Chuvochina, D. W. Waite, C. Rinke, et al., Nat Biotechnol 36:996–1004, 2018, https://doi.org/10.1038/nbt.4229). The scale bars represent amino acid changes. Download FIG S4, PDF file, 0.7 MB. Copyright © 2020 Leu et al. 2020 Leu et al. This content is distributed under the terms of the Creative Commons Attribution 4.0 International license.

### Pathways for energy conservation during AOM in the *Methanoperedenaceae*.

All members of the *Methanoperedenaceae* encode the Fpo complex (FpoABCDHIJ_1_J_2_LMNOF), a homolog of complex I (nuoABCDEFGHIJKLMN), which is hypothesized to oxidize F_420_H_2_ coupled to the reduction of a membrane-bound soluble electron carrier and translocation of two protons out of the cell (Fig. 2 and Fig. S5A) (41, 46). While members of the *Methanosarcinales* and marine ANME-2a are reported to typically use methanophenazine (MP) as their membrane-bound soluble electron carrier, the *Methanoperedenaceae* and ANME-1 have previously been suggested to use menaquinone (MK) based on the annotation of the futalosine pathway for MK biosynthesis in several MAGs representing these lineages (47). Comparative genomic analysis of the 16 *Methanoperedenaceae* MAGs revealed that the futalosine pathway is a conserved feature of all members, except the most basal member, ASW-3 (see below and Data Set S1A). As has previously been suggested by Arshad et al. (24), the larger difference in redox potential between F_420_ (*E*_0_ = –360mV) and MK (*E*_0_ = –80mV [48]) than between F_420_ and MP (*E*_0_ = –165mV [49]) would theoretically allow the Fpo complex to translocate more protons (3H^+^/2e–) out of the cell for every molecule of F_420_ oxidized, giving a higher overall energetic yield from AOM (Fig. S5B).

10.1128/mBio.01325-20.5 FIG S5 Subunit compositions of the Fpo dehydrogenase protein complexes and theoretical bioenergetics of energy metabolism in ANME-2a and *Methanoperedenaceae.* (A) Fpo subunit components for the ANME-2a and ASW-3 genomes (top left) and the other members of the *Methanoperedenaceae* (bottom left). The utilization of different electron carriers shows greater biochemical energetic gains based on more potential proton translocation. The colors orange and green depict *Methanosarcinales*-like and non-*Methanosarcinales*-like subunits. (B) Theoretical redox potential drop when utilizing MP (left) or MK (right) during F420H_2_ and Fd^2–^ oxidation. This is due to differences between the membrane-bound electron carriers’ redox midpoint potential (Em) of –80 mV and –165 mV for MK and MP, respectively (M. Tietze, A. Beuchle, I. Lamla, N. Orth, et al., Chembiochem 4:333–335, 2003, https://doi.org/10.1002/cbic.200390053; Q. H. Tran and G. Unden, Eur J Biochem 251:538–543, 1998, https://doi.org/10.1046/j.1432-1327.1998.2510538.x). Download FIG S5, PDF file, 0.3 MB. Copyright © 2020 Leu et al. 2020 Leu et al. This content is distributed under the terms of the Creative Commons Attribution 4.0 International license.

10.1128/mBio.01325-20.6 FIG S6 Phylogenetic analysis of the Fpo subunits annotated in the *Methanoperedenaceae* genomes. (A) FpoA; (B) FpoB; (C) FpoC; (D) FpoD; (E) FpoH; (F) FpoI; (G) FpoJ1; (H) FpoJ2; (I) FpoK; (J) FpoL; (K) FpoM; (L) FpoN; (M) FpoO. Putative genes recovered from the *Methanoperedenaceae* are indicated in red. The gene trees were inferred using maximum likelihood and support values calculated via nonparametric bootstrapping. Reference genes and the taxonomy are from the GTDB v83 database (D. H. Parks, M. Chuvochina, D. W. Waite, C. Rinke, et al., Nat Biotechnol 36:996–1004, 2018, https://doi.org/10.1038/nbt.4229). Download FIG S6, PDF file, 2.1 MB. Copyright © 2020 Leu et al. 2020 Leu et al. This content is distributed under the terms of the Creative Commons Attribution 4.0 International license.

Phylogenetic analysis of the Fpo complex in the *Methanoperedenaceae* MAGs showed that the FpoKLMNO subunits are homologous to proteins found in MP-utilizing members of the *Methanosarcinales*. The FpoABCDHIJ_1_J_2_ subunits are more similar to those found in microorganisms known to use MK and other quinones, which have more positive redox potentials (Fig. S5 and S6; Data Set S1E) (50). As the latter subunits (specifically FpoH) are responsible for the interaction with the membrane-soluble electron carrier pool (51, 52), this observation provides further support to the use of MK by members of the *Methanoperedenaceae*. To our knowledge, this is the first reported example of a lineage encoding a “hybrid” complex I homolog possessing subunits with homology to those found in phylogenetically diverse microorganisms (Fig. S6). The GoM-Arc-I MAGs appear to possess the MK biosynthesis pathway and a hybrid Fpo complex similar to those of the *Methanoperedenaceae* (Fig. S6), suggesting that the evolutionary adaptation of the lineage to utilize MK occurred prior to the divergence of these two related families. Members of the GoM-Arc-1 clade possess Mcr-like complexes (Fig. S3) and are suggested to use short-chain alkanes, possibly ethane (53, 54). Interestingly, the FpoMNO subunits of the ASW-3 MAG cluster with those of the other members of the *Methanoperedenaceae* family, while their FpoABCDHIJ_1_J_2_KL subunits are most similar to those of the ANME-2a and other members of the *Methanosarcinales* (Fig. S6). While the genes involved in MP biosynthesis are not known, the absence of the MK biosynthesis pathway indicate that ASW-3 likely uses MP. As the most basal lineage of this family, ASW-3 may have adapted to use MP after the evolutionary divergence of the GoM-Arc-I and *Methanoperedenaceae*, although further genomic representation of this lineage is required to verify this hypothesis.

Comparative genomic analyses of the *Methanoperedenaceae* MAGs revealed that none of these genomes encode an Rnf complex, which is hypothesized to reoxidize ferredoxin coupled to the transport of sodium ions out of the cell and the reduction of MP in marine ANME-2a (7, 55) and other methylotrophic methanogens (41, 56, 57). In the absence of this complex, ferredoxins may be reoxidized with a “truncated” Fpo complex, similar to the Fpo complex possessed by Methanosaeta thermophila (58). Alternatively, an electron-confurcating mechanism may be used for the reoxidation of ferredoxin, coenzyme M, and coenzyme B, coupled to the reduction of two F_420_ molecules via a cytoplasmic complex composed of a heterodisulfide reductase (HdrABC) and a F_420_ hydrogenase subunit B (FrhB) (24). The two additional F_420_H_2_ molecules may subsequently be fed back into the Fpo complex, greatly increasing the overall bioenergetic yield (24) (Fig. 2). All of the *Methanoperedenaceae* MAGs have the genetic potential for these alternate strategies for reoxidation of ferredoxin during AOM; however, further experimental validation is required to test these hypotheses.

### Conservation of unique menaquinone: cytochrome *c* oxidoreductases within the *Methanoperedenaceae*.

Five different putative MK:cytochrome *c* oxidoreductase gene clusters (Fig. 1 and 2; Data Set S1A) that are hypothesized to mediate the transfer of electrons out of the cytoplasmic membrane were identified in the *Methanoperedenaceae* MAGs. These gene clusters include a noncanonical bc1/b6f complex adjacent to two hypothetical proteins and two multiheme (6 hemes) *c*-type cytochromes (MHCs; group 1), two clusters where a *b-*type cytochrome is adjacent to a 6-heme MHC (groups 2 and 3), and another two clusters where an NrfD-like transmembrane protein is adjacent to an electron-transferring 4Fe-4S ferredoxin iron-sulfur protein and MHCs (groups 4 and 5) (Fig. 2). These *bc* and NrfD complexes are frequently found in other metal-reducing microorganisms and mediate electron transport from the cytoplasm to the periplasm (59–61).

Most of the 16 *Methanoperedenaceae* MAGs (except CMD-1 and ASW-3) have more than one of these MK:cytochrome oxidoreductase complexes, and 10 have at least four (Fig. 1). ASW-3 is the only MAG not to encode any MK:cytochrome *c* oxidoreductases, which is consistent with its putative use of MP. A gene encoding a cytochrome *b* found to be most similar to that of “*Ca.* Methanohalarchaeum thermophilum” was identified in ASW-3; however, in the absence of a collocated MHC gene, the extracellular electron transfer step for this microorganism is unclear.

Phylogenetic analysis of the membrane-bound subunits of the MK:cytochrome *c* oxidoreductases (Fig. 2), which include the NrfD subunits (from groups 1 and 2) and the *b*-type cytochromes (from groups 3, 4, and 5), showed that they have been potentially laterally transferred from diverse donors (Fig. S7). The *Methanoperedenaceae* NrfD subunits formed independent clusters with sequences from members of the *Dehalococcoidales* family RBG-16-60-22 (group 1) and a single MAG (RBG-16-55-9) from the candidate phylum Bipolaricaulota (group 2) (Fig. S7A). The *b*-type cytochromes of the *Methanoperedenaceae* belong to three distinct clades (Fig. S7B). The *b*-type cytochromes from groups 3 and 4 clustered with proteins from GoM-ArcI, indicating vertical genetic inheritance from an ancestor of these two families, and group 5 proteins clustered with those from the class *Archaeoglobi* (40).

10.1128/mBio.01325-20.7 FIG S7 Phylogenetic analysis of the subunits of the MK:cytochrome oxidoreductases annotated in the *Methanoperedenaceae* MAGs. (A) Analysis of the NrfD subunits; (B) snalysis of the *b*-type cytochromes. Bootstrap values for the maximum-likelihood trees were determined using nonparametric bootstrapping with 100 replicates. The scale bars represent amino acid changes. Download FIG S7, PDF file, 0.7 MB. Copyright © 2020 Leu et al. 2020 Leu et al. This content is distributed under the terms of the Creative Commons Attribution 4.0 International license.

The conservation of multiple conserved laterally transferred MK:cytochrome *c* oxidoreductases in most of the *Methanoperedenaceae* MAGs may contribute to the reported ability of members of the family to reduce a variety of electron acceptors with a range of redox potentials that include Fe(III) oxide reduction (–100mV to 100 mV) (62), nitrate (+433 mV) (24), and Mn(IV) (+380 mV) (36). Transcriptomic analyses have shown that different MK:cytochrome *c* oxidoreductases are expressed in different species of the genus “*Ca*. Methanoperedens” during AOM coupled to the reduction of Fe(III) oxides (9), Mn(IV) oxides (12), and nitrate (15, 24). A similar phenomenon has been observed for the species Geobacter sulfurreducens, where different extracellular electron pathways were used when different electron acceptors were reduced (63).

### Potential electron acceptors used by the *Methanoperedenaceae*.

Annotation of the *Methanoperedenaceae* MAGs revealed a wide array of genes associated with previously undescribed respiratory strategies for the family that appear to have been acquired via LGT. Principally, these are putative terminal oxidoreductase complexes belonging to the complex-iron–sulfur–molybdenum (CISM) superfamily that were absent in the genomes of related archaeal lineages (Fig. 3). These complexes are composed of a catalytic subunit, an iron-sulfur protein, and a membrane-bound subunit and facilitate the transfer of electrons between the electron acceptor/donor and the MK pool (Fig. 2).

As previously reported, the MAGs M.Nitro, BLZ2, and IPS-1 encode respiratory nitrate reductases that are part of the CISM superfamily and have been demonstrated to mediate AOM coupled to nitrate reduction (15, 24, 29). Based on phylogenetic analysis (Fig. 3), genes encoding cytoplasmic nitrite oxidoreductases (NxrA) were identified in the IPS-1, BLZ2, and M.Nitro MAGs, and a nitrate reductase closely related to NarG proteins was identified in the BLZ2 MAG. Of the *Methanoperedenaceae* MAGs, only the M.Nitro and BLZ2 MAGs possess a putative nitrite reductase (NrfA) for DNRA. The M.Ferri MAG encodes an assimilatory nitrate reductase (NarB/NasA) most similar to a protein encoded by “*Candidatus* Magnetobacterium casensis” (Fig. 3). However, in the absence of an annotated nitrite reductase in the M.Ferri MAG, the potential of this microorganism for assimilatory nitrate reduction is unclear.

Multiple MAGs (ASW-2,3,5,6 and Mn-2) were also found to encode putative selenate reductases (SrdA) (Fig. 3), suggesting their ability for Se(VI)-dependent AOM. Recently, a bioreactor enrichment of a member of the genus “*Ca.* Methanoperedens” exhibited AOM activity when nitrate was replaced with selenate (26). However, as no meta-omic analyses was conducted for the community, it is unclear if the dominant “*Ca.* Methanoperedens” possessed a putative selenate reductase or if it was directly responsible for the observed selenate reduction.

The ASW-1 and ASW-3 MAGs encode a putative sulfur reductase (SreABC). This annotation is supported by its phylogenetic clustering of the catalytic subunit with SreA from Aquifex aeolicus (Fig. 3), which has been shown to reduce elemental sulfur, as well as tetrathionate and polysulfide (64). This is the first genomic evidence suggesting that members of the *Methanoperedenaceae* may be involved in respiratory sulfur-dependent AOM and warrants further investigation. ANME-1 has been proposed to couple AOM to the reduction of polysulfide in a biogenic hydrocarbon seep sediment, but this was based on the annotation and high expression of a putative sulfide: quinone oxidoreductase (SQR) (65). Genes for dissimilatory sulfate reduction pathways were absent in the *Methanoperedenaceae* MAGs, consistent with other ANME lineages (66). MGW-1 was recently speculated to directly couple AOM to sulfate reduction by utilizing assimilatory sulfate reduction pathways. This hypothesis was based on the lack of large MHCs or identifiable alternate electron acceptor complexes encoded in the MAG (30). Several of the *Methanoperedenaceae* MAGs, and those of other ANME lineages, contain candidate genes associated with assimilatory sulfate reduction, but a dissimilatory role for these has not been shown (66).

The M.Nitro MAG encodes two putative reductases belonging to the arsenate reductase (ArrA) and arsenite oxidase (ArxA) group (Fig. 3). The BLZ2, ASW-1, ASW-4, and IPS-1 MAGs also encode reductases that cluster with the M.Nitro ArxA-like sequence. The ArxA protein has been found to be capable of both arsenite oxidation and arsenate reduction (67), which would allow the *Methanoperedenaceae* possessing these ArxA-like proteins to utilize arsenate as a terminal electron acceptor. Proteins encoded by the ASW-3 and “*Candidatus* Acetothermum autotrophicum” (68) (Fig. 3) form a deep branching clade adjacent to the ArxA and ArrA groups, suggesting that these species might also have the potential to respire on arsenic compounds. It is noteworthy that the ASW-1, -3, and -4 MAGs were recovered from a Bangladesh arsenic-contaminated groundwater sample (Table 1), indicating a role for LGT in their niche-specific adaptation. The possibility of AOM coupled to arsenate [As(V)] reduction has important environmental implications given the wide distribution of arsenic in nature, including subsurface drinking water aquifers (69), and the toxicity and mobility of its reduced form, arsenite [As(III)] (70, 71). Arsenic reduction and mobilization have been linked to an inflow of organic carbon in contaminated aquifers where methane (∼1 mM) and arsenate cooccur (72, 73).

Additional putative oxidoreductase clades that are not closely associated with any well-characterized CISM proteins were also found in the *Methanoperedenaceae* MAGs. This includes two proteins encoded by the ASW-3 and ASW-6 MAGs that cluster with a protein of unknown function from a “*Candidatus* Brocadiales” MAG (74) and the CMD-1 protein that clusters with a protein from “*Candidatus* Brocadia fulgida,” an ammonium-oxidizing and nitrite-reducing microorganism (75). In general, given the large range of substrates utilized by the CISM superfamily and the few biochemically characterized proteins, the predicted functions of all those annotated in the *Methanoperedenaceae* require empirical verification. Nonetheless, the range of putative CISM superfamily proteins encoded by members of the family likely indicates diverse respiratory strategies that remain to be characterized.

### Diversity of the MHCs in the *Methanoperedenaceae*.

Members of the *Methanoperedenaceae* possess a diverse repertoire of MHCs which have been suggested to facilitate the transfer of electrons from the reoxidation of MK to metal oxides (9, 10, 76) or direct interspecies electron transfer (DIET) to a syntrophic partner. Analyses of the *Methanoperedenaceae* revealed that they possess between 3 (MGW-1) and 49 (IPS-1) MHCs (containing at least three CXXCH motifs), with an average of 26, the highest average of any archaeal family (Data Sets S1F and S1G). Notably, relatively high numbers of MHCs per genome are almost exclusively found in microorganisms associated with DIET, metal and/or sulfur reduction, such as the *Geobacteraceae* (77) (≤87 MHCs), *Shewanellaceae* (78) (≤63 MHCs), *Desulfurivibrionaceae* (20), *Desulfuromonadaceae* (20), and *Defferisomataceae* (79) (≤50 MHCs) (Data Set S1G). Interestingly, 7 of the 16 members of the *Methanoperedenaceae* encode MHCs with more than 50 heme binding sites (ASW-5, ASW-6, BLZ2, HGW-1, M.ferri, Mn-1, and Mn-2), with the 113-heme MHC encoded by Mn-2 being the largest identified in any microorganism (Data Set S1F).

The 414 putative MHCs identified in the *Methanoperedenaceae* MAGs clustered into 82 orthologous protein families (Fig. S8). Only one protein family (OG0000252) included at least one MHC from each member, which suggests low conservation of these genes within the *Methanoperedenaceae*. Out of the 82 MHC protein families, 14 were identified in at least eight of the 16 MAGs, with five of these found within the conserved MK:cytochrome *c* oxidoreductase clusters. A lack of conservation of MHCs is also observed for anaerobic metal-respiring genus *Geobacter* organisms, where 14% of the MHCs encoded in six analyzed genomes were found to be conserved (60). Thirty-nine of the 82 MHC protein families had significant hits (1e−20, ≥50% AAI) to homologs from diverse lineages across the bacterial and archaeal domains in the GTDB89 database, indicating potential LGT of these genes (Fig. S9). These lineages notably included the metal-reducing *Geobacteraceae* and *Shewanellaceae*, along with the alkane-oxidizing *Archaeoglobaceae*, *Methylomirabilota* (NC10), and other ANME lineages (Fig. S9).

10.1128/mBio.01325-20.8 FIG S8 Abundance profiles for the MHC-orthologous protein families annotated in the *Methanoperedenaceae* MAGs. Download FIG S8, PDF file, 0.4 MB. Copyright © 2020 Leu et al. 2020 Leu et al. This content is distributed under the terms of the Creative Commons Attribution 4.0 International license.

10.1128/mBio.01325-20.9 FIG S9 Network analysis of MHC-orthologous protein families in *Methanoperedenaceae*. Each cluster represents related MHCs. The color of the nodes represents the taxonomic lineage based on GTDB classification. The size of the nodes represents the number of CXXCH heme binding motifs identified in the proteins. The thicknesses of the lines represent amino acid identity between the two nodes. The shaded boxes represent the orthologous protein families. Download FIG S9, PDF file, 0.5 MB. Copyright © 2020 Leu et al. 2020 Leu et al. This content is distributed under the terms of the Creative Commons Attribution 4.0 International license.

### Putative functions of MHCs in the *Methanoperedenaceae*.

Very few of the *Methanoperedenaceae* MHCs could be associated with a specific function. Two orthologous groups were annotated as nitrite: ammonium oxidoreductases (NrfA), with homologs identified in bacterial MAGs classified as *Anaerolineales* (OG0004545; ≥66.3% AAI), and the candidate phylum UBP4 (OG0012490, 64.56% AAI). Several MHCs were also identified as part of the MK:cytochrome *c* oxidoreductase clusters, with homologs observed in members of the archaeal family *Archaeoglobaceae* (OG001557, OG000137, OG0001550; ≥57.3% AAI) (Fig. S9). MHC/S-layer fusion proteins were suggested to mediate the transfer of electrons across the S-layer for marine ANME-2 (7) and were relatively highly expressed by “*Ca.* Methanoperedens manganicus” and “*Ca.* Methanoperedens manganireducens” during AOM coupled to Mn(IV) reduction (12). Conversely, only low expression of MHC/S-layer protein genes borne by “*Ca.* Methanoperedens ferrireducens” was observed during AOM coupled to Fe(III) reduction (9). In addition, despite all the *Methanoperedenaceae* MAGs containing S-layer proteins, five do not encode MHC proteins with an S-layer domain (ASW-3, CMD-1, CMD-2, HGW-1, and MGW-1), indicating alternative mechanisms for electron transfer across the S-layer to extracellular MHCs for these species.

Predicted extracellular MHCs are hypothesized to facilitate the final transfer of electrons from the *Methanoperedenaceae* to metal oxides (9). Interestingly, “*Ca.* Methanoperedens manganicus” and “*Ca.* Methanoperedens manganireducens” showed differential expression patterns in the complement of shared extracellular MHCs during AOM coupled to Mn(IV) reduction. In addition, no orthologs for the two MHCs highly transcribed by “*Ca*. Methanoperedens ferrireducens” during AOM coupled to Fe(III) reduction (9) were identified in other members of the *Methanoperedenaceae* (OG0011636 and OG0003254) (Fig. S8), suggesting that BLZ2 utilizes a different MHC for iron reduction linked to AOM (10). These observations suggest that the *Methanoperedenaceae* can utilize multiple mechanisms for the reduction of similar metal oxides. Differential expression of conserved MHCs linked to extracellular electron transfer was also observed for different *Geobacteraceae* species enriched on electrodes when they were exposed to the same surface redox potential (80). As suggested for members of the *Geobacteraceae*, the large MHC repertoire possessed by the *Methanoperedenaceae* may enable adaptation to the use of a range of terminal electron acceptors.

This study has substantially improved the genome coverage of the *Methanoperedenaceae*. Comparative genomic analysis of this lineage highlights a metabolic plasticity not found in other ANME clades. The subsequent ability of members of the family to adapt to the use of terminal electron acceptors across a range of redox potentials likely contributes to their success in diverse environments (Table 1). Notably, based on the genome tree (Fig. 1) and the lack of conservation of MHCs (Fig. S8), the acquisition of these genes is not congruent with the genome-based phylogeny of the family, suggesting niche-specific adaptations as the main driver for these LGT events. While further studies are necessary to verify the general physiology and energy conservation mechanisms of the *Methanoperedenaceae* in different environments, this study provides genomic evidence that members of the family may play key roles in coupling cycling of carbon with selenate, sulfur, and arsenic in addition to nitrate and metal oxides. Continued sequencing and characterization of this lineage will reveal the full extent of their metabolic versatility and influence on global biogeochemical cycles.

## MATERIALS AND METHODS

### Recovery of the genomes from SRA.

The NCBI sequence read archive (SRA [81]) was accessed on the 22nd of March 2017, and 14,516 data sets classified as environmental metagenomes were downloaded. The metagenomic data sets were screened using GraftM (32) to search for 16S rRNA and *mcrA* gene sequences similar to those from members of the *Methanoperedenaceae*. For data sets for which members of the family were detected, all paired-end read sets were trimmed and quality filtered using PEAT v1.2.4 (82). For genomes, CMD-1 and CMD-2 and NCBI accession number SRR5161805 and SRR5161795 reads were coassembled using metaSPAdes version 3.10.0 using the default parameters (83). For the ASW genomes, NCBI accession number SRR1563167, SRR1564103, SRR1573565, and SRR1573578 reads were coassembled using metaSPAdes version 3.10.0, with default parameters (83). Mapping of quality reads was performed using BamM v1.7.3, with default parameters (https://github.com/Ecogenomics/BamM). Metagenomic assembled genomes were recovered from the assembled metagenomes using uniteM v0.0.14 (https://github.com/dparks1134/UniteM). The *Methanoperedenaceae* MAGs were further refined by reassembling the mapped quality trimmed reads with SPAdes using the –careful and –trusted contig settings. Additional scaffolding and resolving ambiguous bases of the MAGs was performed using the “roundup” mode of FinishM, v0.0.7 (https://github.com/wwood/finishm). The completeness and contamination rates of the population bins were assessed using CheckM v1.0.11 (33) with the “lineage wf” command.

### Functional annotation.

For all MAGs, open reading frames (ORFs) were called and annotated using Prokka v.1.12 (84). Additional annotation was performed using the blastp “verysensitive” setting in Diamond v0.9.18 (https://github.com/bbuchfink/diamond.git) against UniRef100 (accessed September 2017) (85), clusters of orthologous groups (COG) (86), Pfam 31 (87), and TIGRfam (released January 2014) (88). ORFs were also diamond blastp searched against Uniref100 (accessed September 2017) containing proteins with KO identifiers (IDs). The top hit for each gene with an E value of <1e−3 was mapped to the KO database (89) using the UniProt ID mapping files. Genes of interest were further verified using the NCBI’s conserved domain search to identify a conserved motif(s) present within the gene (90). Psortb v3.0 (91) was used to predict subcellular localization of the putative proteins. Pred-Tat was used to predict putative signal peptides (92). Putative MHCs were identified by ORFs possessing ≥3 CXXCH motifs. Putative MHCs were subsequently searched for cytochrome *c*-type protein domains using hmmsearch (HMMER v.3.1) (93) with PfamA (94).

### Construction of genome trees.

The archaeal genome tree was constructed using GTDB-Tk (GTDBtk v0.2.2; https://github.com/Ecogenomics/GTDBTk/releases) with a concatenated set of 122 archaeon-specific conserved marker genes inferred from genomes available in the NCBI database (NCBI RefSeq release 83) (34). Marker genes were identified and aligned in each genome using HMMER v.3.1 (93) and concatenated, and trees were constructed using FastTree v.2.1.8 (95) with the WAG+GAMMA models. Support values were determined using 100 nonparametric bootstrapping with GenomeTreeTK. The trees were visualized using ARB (96) and formatted using Adobe Illustrator (Adobe, USA).

### Construction of 16S rRNA gene tree.

The 16S rRNA gene was identified in MAGs and used to infer taxonomic assignment of the population genome implementing the SILVA 16S rRNA gene database (version 132). Sequences were aligned with 426 16S rRNA gene sequences retrieved from the SILVA database using SSU-align v0.1 (97). The phylogenetic tree was constructed using FastTree v2.1.8 (95) with the Generalised Time-Reversible and GAMMA models. Support values were determined using 100 nonparametric bootstrapping. The trees were visualized using ARB (96) and formatted using Adobe Illustrator.

### Calculation of amino acid identity.

The *Methanoperedenaceae* MAGs identified in this study were compared to publicly available genomes of the family. Average amino acid identity (AAI) between the genomes was calculated using orthologous genes identified through reciprocal best BLAST hits using compareM v0.0.5 (https://github.com/dparks1134/CompareM).

### Identification of orthologous proteins.

Homologous proteins across MAGs of all the *Methanoperedenaceae*, GoM-Arc I, ANME-2a, and ANME-2c were identified with OrthoFinder (98) v2.3.3 using default parameters. Gene counts of orthologous groups containing MHCs were used as input for a heatmap using the pheatmap package in R, and hierarchical clustering was performed using ward.D2 (99).

### Construction of gene trees.

Genes of interest in the *Methanoperedenaceae* MAGs were compared against proteins from the GTDB v83 database (34) using the genetreetk “blast” command to identify closely related sequences. For the generation of the gene tree for catalytic subunits of the CISM superfamily, curated protein sequences were also added in the analysis. Accession numbers and amino acid sequences are included in Data Set S1B. For the generation of the gene tree for the catalytic subunits of the group 1 and group 3 NiFe dehydrogenase, curated sequences from the work of Greening et al. (100) were included in the analysis. Accession numbers and amino acid sequences can be found in Data Set S1C. The sequences were subsequently aligned using mafft v7.221 (101) with the -auto function, and the alignment was trimmed using the trimal v1.2 (https://github.com/scapella/trimal) “-automated1” option. A phylogenetic tree was constructed using RAxML v8.2.9 (102) with the following parameters: raxmlHPC-PTHREADS-SSE3 -T 30 -m PROTGAMMALG -p 12345. Bootstrap values were calculated via nonparametric bootstrapping with 100 replicates. The trees were visualized using ARB (96) or iToL (103) and formatted using Adobe Illustrator (Adobe, USA).

### Network analysis of MHCs.

Putative MHCs from the GTDB v89 database were identified by ORFs possessing ≥3 CXXCH motifs. Putative MHCs were subsequently searched for cytochrome *c*-type protein domains using hmmsearch (HMMER v.3.1) (93) with PfamA (94). Proteins from each *Methanoperedenaceae* orthogroup were subjected to a blast search against the GTDB v89 MHC protein database using DIAMOND with an E value cutoff of 1e–20 and ≥50% AAI. The result was visualized in Cytoscape v3.7.1, with clusters that contained only, or no, *Methanoperedenaceae* homologs removed.

### Data availability.

The genomes assembled in this study have been deposited in the NCBI database under the accession numbers SAMN10961276 to SAMN10961283.
