# Supplementary material for: Lateral Gene Transfer Drives Metabolic Flexibility in the Anaerobic Methane-Oxidizing Archaeal Family Methanoperedenaceae
Source: mBio. 2020 Jun 30;11(3):e01325-20. doi: 10.1128/mBio.01325-20 (PMC7327174; doi:10.1128/mBio.01325-20)
Supplement: FIG S1 [file mBio.01325-20-sf001.pdf]

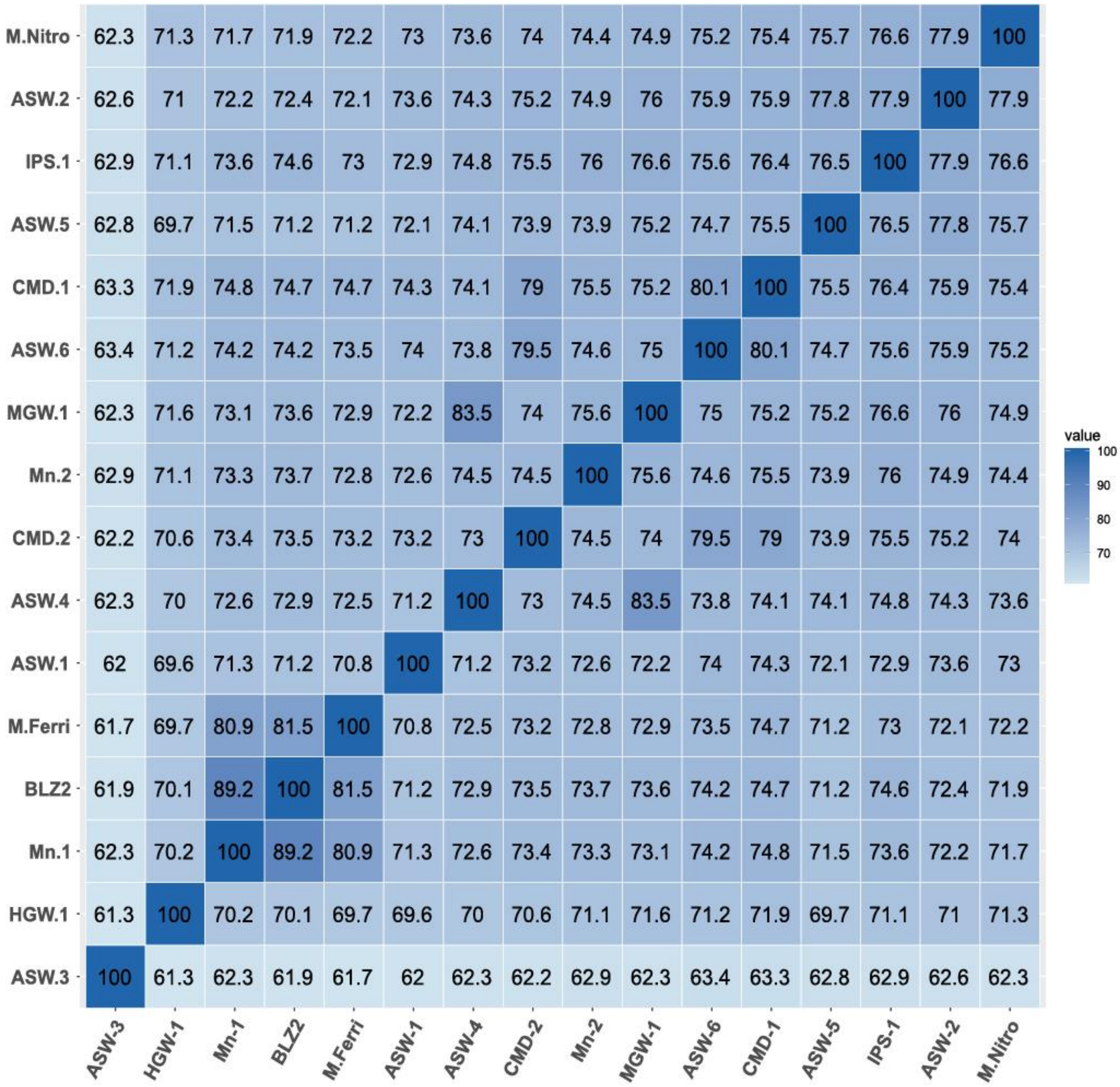


**Figure S1. Average amino acid identity (AAI%) for the *Methanoperedenaceae* genomes.** AAI was calculated between each pair of genomes using CompareM.
