# Supplementary material for: Lateral Gene Transfer Drives Metabolic Flexibility in the Anaerobic Methane-Oxidizing Archaeal Family Methanoperedenaceae
Source: mBio. 2020 Jun 30;11(3):e01325-20. doi: 10.1128/mBio.01325-20 (PMC7327174; doi:10.1128/mBio.01325-20)
Supplement: FIG S2 [file mBio.01325-20-sf002.pdf]

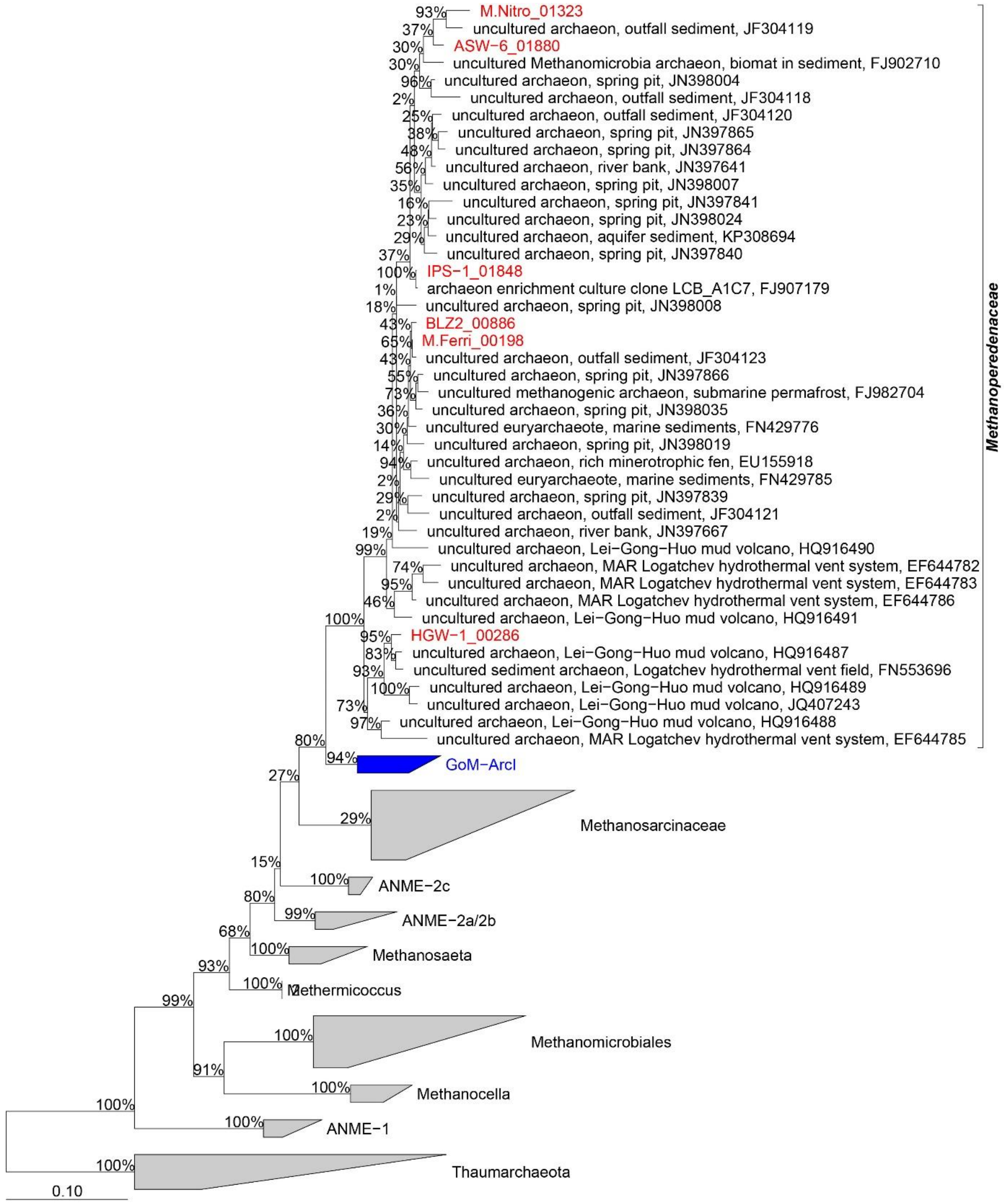

Figure S2. 16S rRNA gene based phylogenetic placement of the *Methanoperedenaceae* MAGs. The 16S rRNA genes extracted from the *Methanoperedenaceae* MAGs from this study are highlighted in red. Support values calculated via non-parametric bootstrapping. The scale bar represents changes per nucleotide position.
