# Supplementary material for: Lateral Gene Transfer Drives Metabolic Flexibility in the Anaerobic Methane-Oxidizing Archaeal Family Methanoperedenaceae
Source: mBio. 2020 Jun 30;11(3):e01325-20. doi: 10.1128/mBio.01325-20 (PMC7327174; doi:10.1128/mBio.01325-20)
Supplement: FIG S3 [file mBio.01325-20-sf003.pdf]

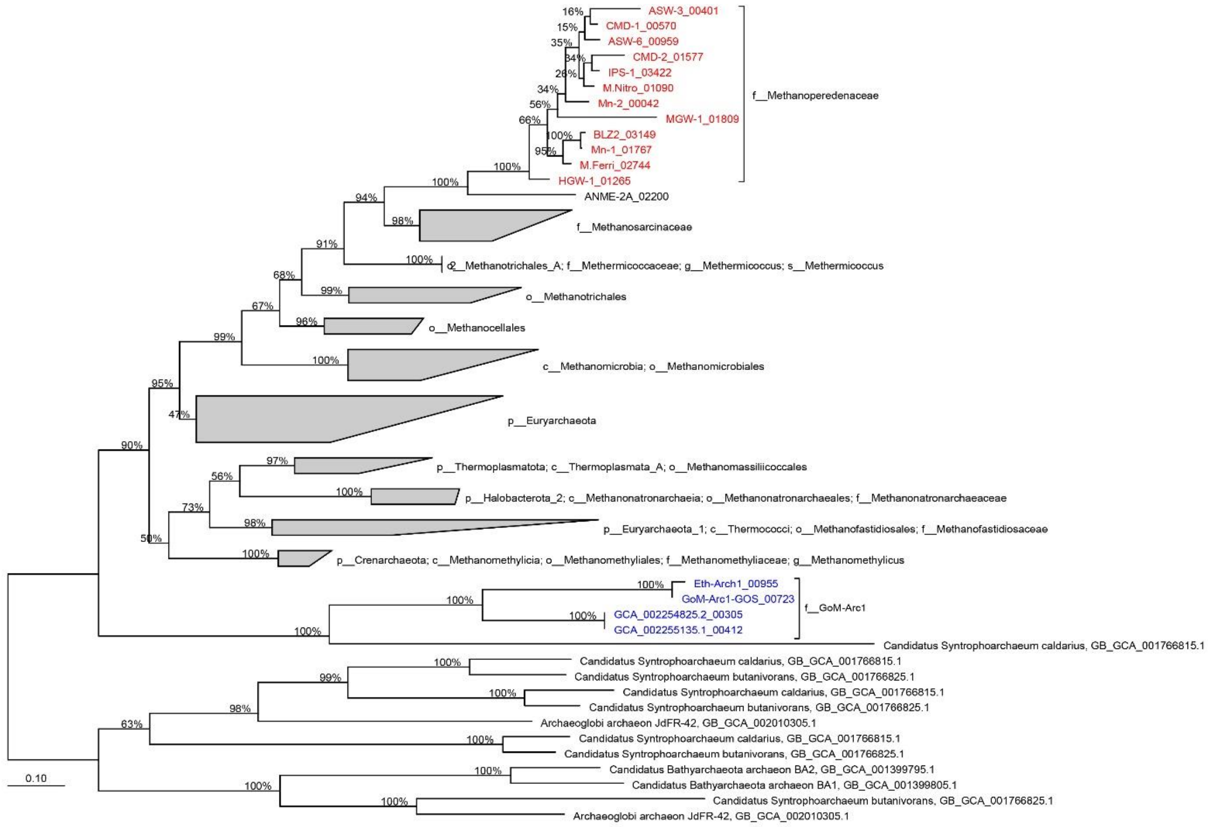

Figure S3. Phylogenetic analysis of methyl-coenzyme reductase subunit A (McrA). Putative genes recovered from the *Methanoperedenaceae* are highlighted in red. The gene tree was inferred using maximum likelihood and support values calculated via non-parametric bootstrapping. The scale bar represents amino acid changes.
