# Supplementary material for: Lateral Gene Transfer Drives Metabolic Flexibility in the Anaerobic Methane-Oxidizing Archaeal Family Methanoperedenaceae
Source: mBio. 2020 Jun 30;11(3):e01325-20. doi: 10.1128/mBio.01325-20 (PMC7327174; doi:10.1128/mBio.01325-20)
Supplement: FIG S4 [file mBio.01325-20-sf004.pdf]

A.

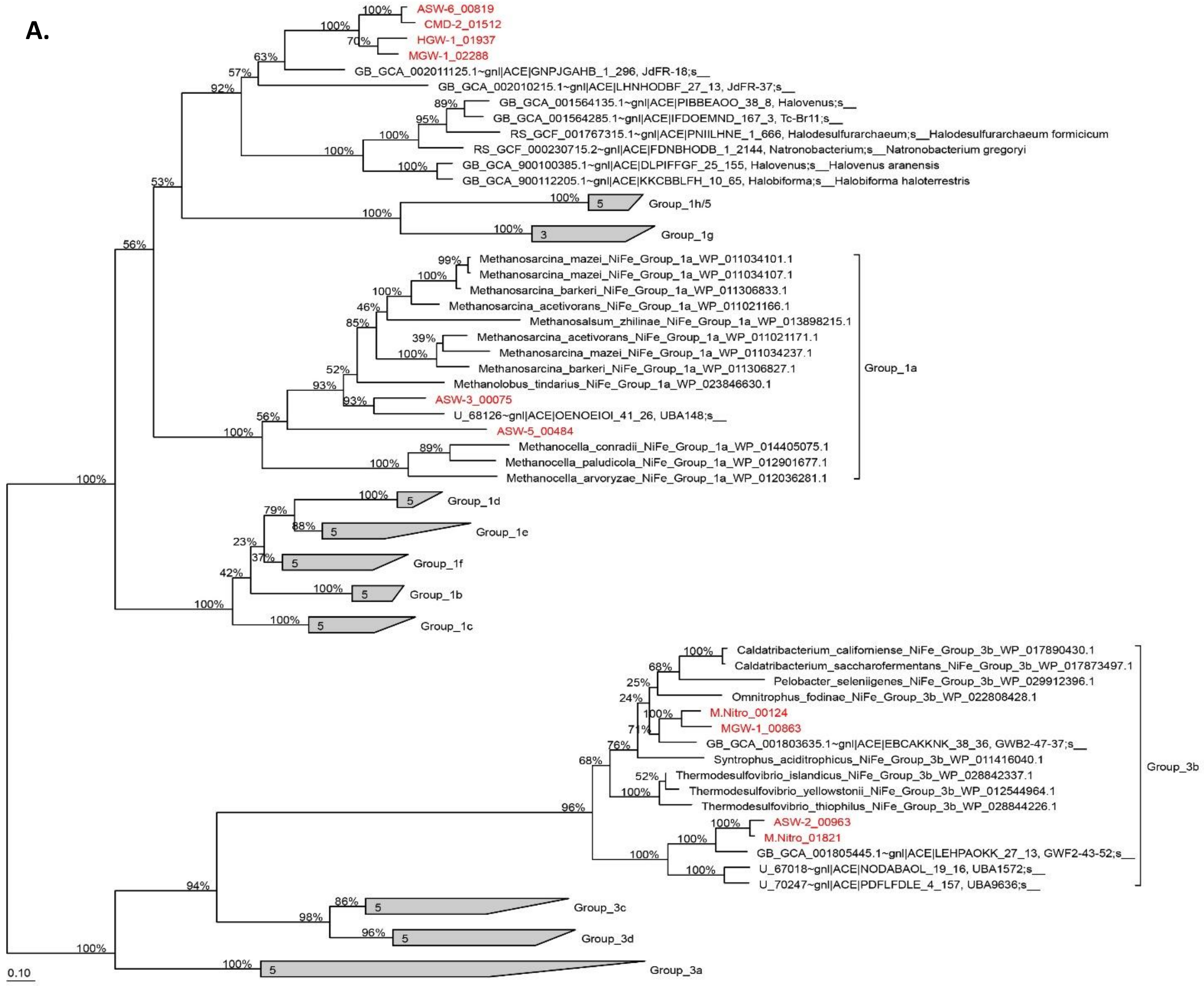
ASW-6_00819
CMD-2_01512
HGW-1_01937
MGW-1_02288
GB_GCA_002011125.1~gnl|ACE|GNPJGAHB_1_296, JdFR-18;s__
GB_GCA_002010215.1~gnl|ACE|LHNHODBF_27_13, JdFR-37;s__
GB_GCA_001564135.1~gnl|ACE|PIBBEAOO_38_8, Halovenus;s__
GB_GCA_001564285.1~gnl|ACE|IFDOEMND_167_3, Tc-Br11;s__
RS_GCF_001767315.1~gnl|ACE|PNIILHNE_1_666, Halodesulfurarchaeum;s__Halodesulfurarchaeum formicicum
RS_GCF_000230715.2~gnl|ACE|FDNBHODB_1_2144, Natronobacterium;s__Natronobacterium gregoryi
GB_GCA_900100385.1~gnl|ACE|DLPIFFGF_25_155, Halovenus;s__Halovenus aranensis
GB_GCA_900112205.1~gnl|ACE|KKCBBLFH_10_65, Halobiforma;s__Halobiforma haloterrestris
Group_1h/5
Group_1g
Methanosarcina_mazei_NiFe_Group_1a_WP_011034101.1
Methanosarcina_mazei_NiFe_Group_1a_WP_011034107.1
Methanosarcina_barkeri_NiFe_Group_1a_WP_011306833.1
Methanosarcina_acetivorans_NiFe_Group_1a_WP_011021166.1
Methanosalsum_zhilinae_NiFe_Group_1a_WP_013898215.1
Methanosarcina_acetivorans_NiFe_Group_1a_WP_011021171.1
Methanosarcina_mazei_NiFe_Group_1a_WP_011034237.1
Methanosarcina_barkeri_NiFe_Group_1a_WP_011306827.1
Methanolobus_tindarius_NiFe_Group_1a_WP_023846630.1
ASW-3_00075
U_68126~gnl|ACE|OENOEIOI_41_26, UBA148;s__
ASW-5_00484
Methanocella_conradii_NiFe_Group_1a_WP_014405075.1
Methanocella_paludicola_NiFe_Group_1a_WP_012901677.1
Methanocella_arvoryzae_NiFe_Group_1a_WP_012036281.1
Group_1a
Group_1d
Group_1e
Group_1f
Group_1b
Group_1c
Caldatribacterium_californiense_NiFe_Group_3b_WP_017890430.1
Caldatribacterium_saccharofermentans_NiFe_Group_3b_WP_017873497.1
Pelobacter_seleniigenes_NiFe_Group_3b_WP_029912396.1
Omnitrophus_fodinae_NiFe_Group_3b_WP_022808428.1
M.Nitro_00124
MGW-1_00863
GB_GCA_001803635.1~gnl|ACE|EBCAKKNK_38_36, GWB2-47-37;s__
Syntrophus_aciditrophicus_NiFe_Group_3b_WP_011416040.1
Thermodesulfovibrio_islandicus_NiFe_Group_3b_WP_028842337.1
Thermodesulfovibrio_yellowstonii_NiFe_Group_3b_WP_012544964.1
Thermodesulfovibrio_thiophilus_NiFe_Group_3b_WP_028844226.1
ASW-2_00963
M.Nitro_01821
GB_GCA_001805445.1~gnl|ACE|LEHPAOKK_27_13, GWF2-43-52;s__
U_67018~gnl|ACE|NODABAOL_19_16, UBA1572;s__
U_70247~gnl|ACE|PDFLFDLE_4_157, UBA9636;s__
Group_3b
Group_3c
Group_3d
Group_3a
0.10

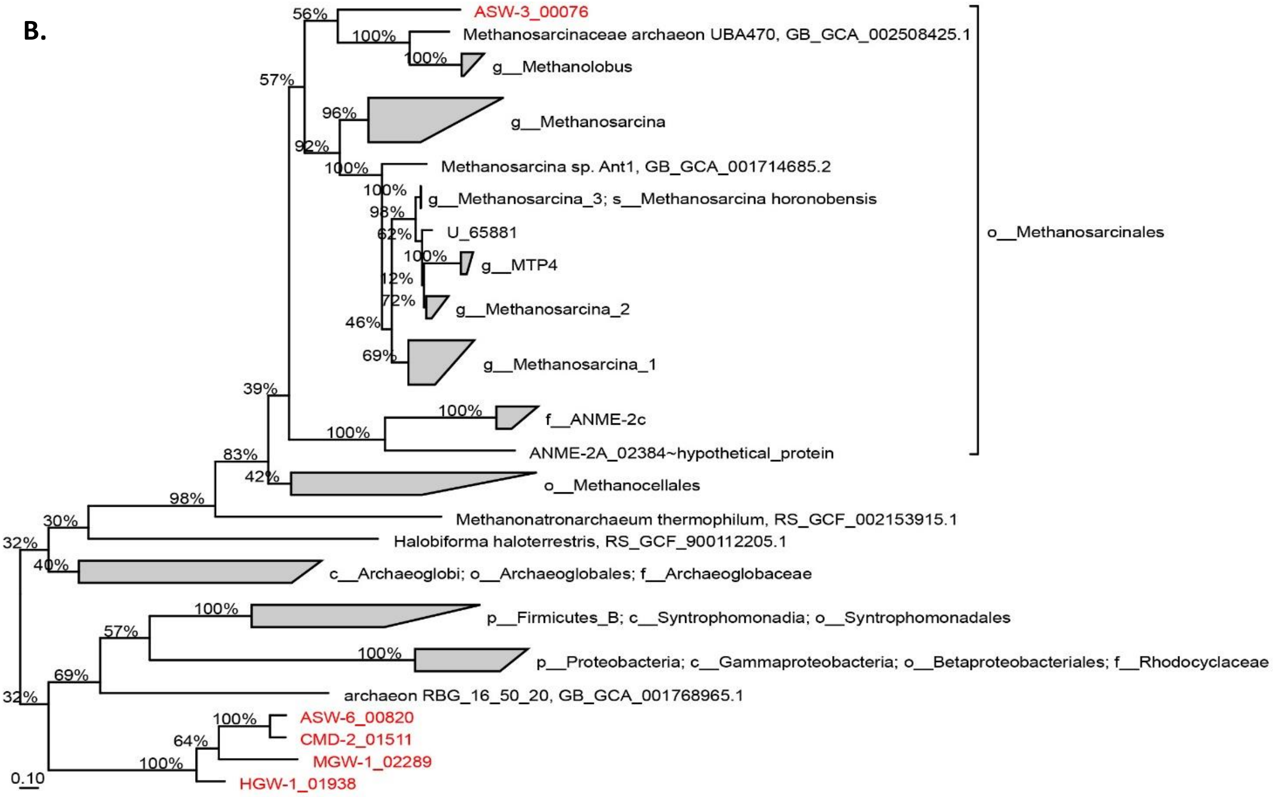

Figure S4. Phylogenetic analysis of the subunits of the NiFe hydrogenases annotated in the *Methanoperedenaceae* genomes. A. Analysis of the catalytic subunits of the energy-converting NiFe hydrogenases. **B.** Analysis of the b-type cytochrome in the Group 1 NiFe hydrogenases. Putative genes recovered from the *Methanoperedenaceae* are highlighted in red. The gene trees were inferred using maximum likelihood and support values calculated via non-parametric bootstrapping. The reference sequences of Group 1 and Group 3 NiFe hydrogenases were acquired from Greening *et al*., (C. Greening, A. Biswas, C. R. Carere, C. J. Jackson, M. C. Taylor, M. B. Stott, G. M. Cook and S. E. Morales, ISME J 10: 761-777, 2016, https://doi.org/10.1038/ismej.2015.153) and the GTDB v83 reference sequences (D.H. Parks, M. Chuvochina, D. W. Waite, C. Rinke, A. Skarshewski, P.-A. Chaumeil and P. Hugenholtz, Nat Biotechnol 36: 996-1004, 2018, https://doi.org/10.1038/nbt.4229). The scale bar represents amino acid changes.
