# Supplementary material for: Lateral Gene Transfer Drives Metabolic Flexibility in the Anaerobic Methane-Oxidizing Archaeal Family Methanoperedenaceae
Source: mBio. 2020 Jun 30;11(3):e01325-20. doi: 10.1128/mBio.01325-20 (PMC7327174; doi:10.1128/mBio.01325-20)
Supplement: FIG S5 [file mBio.01325-20-sf005.pdf]

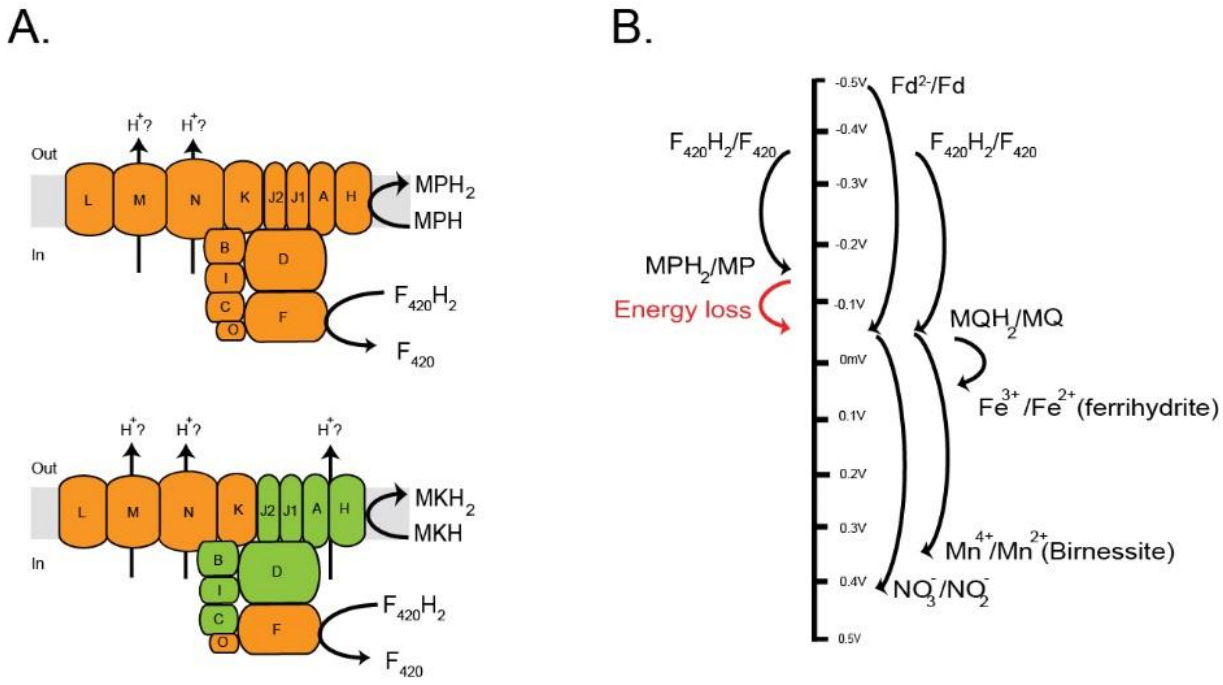

Figure S5. Subunit compositions of the Fpo dehydrogenase protein complexes and theoretical bioenergetics of energy metabolism in ANME-2a and *Methanoperedenaceae*. **A.** Fpo subunit components for the ANME-2a and ASW-3 genomes (top left) and the other members of the *Methanoperedenaceae* (bottom left). The utilization of different electron carriers shows greater biochemical energetic gains based on more potential proton translocation. The colours orange and green depict *Methanosarcinales*-like and non-*Methanosarcinales*-like subunits. **B.** Theoretical redox potential drop when utilizing MP (left) or MK (right) during $F_{420}H_2$ and Fd^{2-} oxidation. This is due to differences between the membrane-bound electron carriers' redox midpoint potential (E_m) of -80mV and -165mV for MK and MP, respectively (M., Tietze, A. Beuchle, I. Lamla, N. Orth, M. Dehler, G. Greiner and U. Beifuss, Chembiochem 4: 333-335, 2003, https://doi.org/10.1002/cbic.200390053; Q.H. Tran and G. Unden, Eur. J. of Biochem. 251: 538-543, 1998, https://doi.org/10.1046/j.1432-1327.1998.2510538.x).
