# Supplementary material for: Lateral Gene Transfer Drives Metabolic Flexibility in the Anaerobic Methane-Oxidizing Archaeal Family Methanoperedenaceae
Source: mBio. 2020 Jun 30;11(3):e01325-20. doi: 10.1128/mBio.01325-20 (PMC7327174; doi:10.1128/mBio.01325-20)
Supplement: FIG S6 [file mBio.01325-20-sf006.pdf]

A.

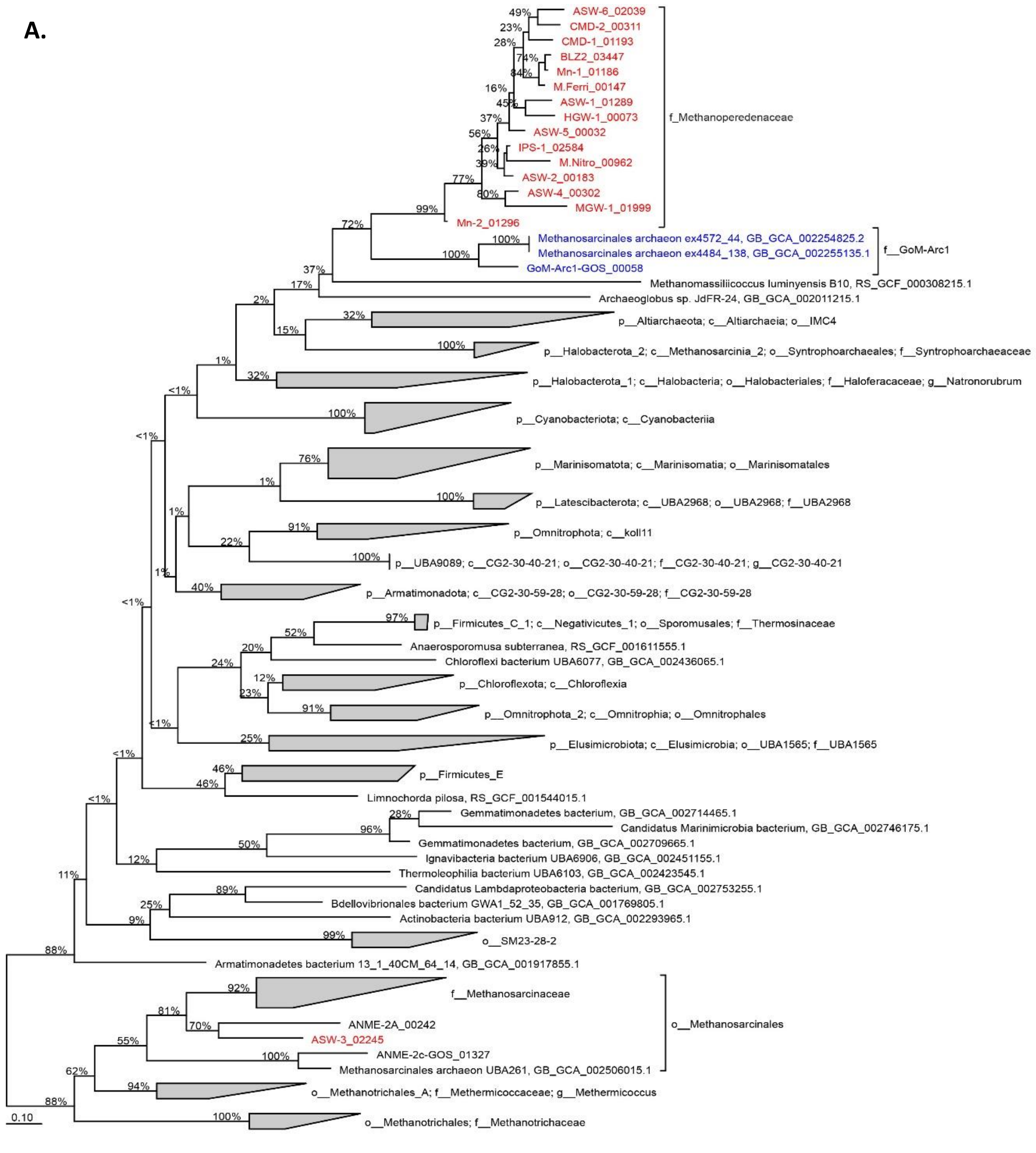
ASW-6_02039
CMD-2_00311
CMD-1_01193
BLZ2_03447
Mn-1_01186
M.Ferri_00147
ASW-1_01289
HGW-1_00073
ASW-5_00032
IPS-1_02584
M.Nitro_00962
ASW-2_00183
ASW-4_00302
MGW-1_01999
Mn-2_01296
f_Methanoperedenaceae
Methanosarcinales archaeon ex4572_44, GB_GCA_002254825.2
Methanosarcinales archaeon ex4484_138, GB_GCA_002255135.1
GoM-Arc1-GOS_00058
f__GoM-Arc1
Methanomassiliicoccus luminyensis B10, RS_GCF_000308215.1
Archaeoglobus sp. JdFR-24, GB_GCA_002011215.1
p__Altiarchaeota; c__Altiarchaeia; o__IMC4
p__Halobacterota_2; c__Methanosarcinia_2; o__Syntrophoarchaeales; f__Syntrophoarchaeaceae
p__Halobacterota_1; c__Halobacteria; o__Halobacteriales; f__Haloferacaceae; g__Natronorubrum
p__Cyanobacteriota; c__Cyanobacteriia
p__Marinisomatota; c__Marinisomatia; o__Marinisomatales
p__Latescibacterota; c__UBA2968; o__UBA2968; f__UBA2968
p__Omnitrophota; c__koll11
p__UBA9089; c__CG2-30-40-21; o__CG2-30-40-21; f__CG2-30-40-21; g__CG2-30-40-21
p__Armatimonadota; c__CG2-30-59-28; o__CG2-30-59-28; f__CG2-30-59-28
p__Firmicutes_C_1; c__Negativicutes_1; o__Sporomusales; f__Thermosinaceae
Anaerosporomusa subterranea, RS_GCF_001611555.1
Chloroflexi bacterium UBA6077, GB_GCA_002436065.1
p__Chloroflexota; c__Chloroflexia
p__Omnitrophota_2; c__Omnitrophia; o__Omnitrophales
p__Elusimicrobiota; c__Elusimicrobia; o__UBA1565; f__UBA1565
p__Firmicutes_E
Limnochorda pilosa, RS_GCF_001544015.1
Gemmatimonadetes bacterium, GB_GCA_002714465.1
Candidatus Marinimicrobia bacterium, GB_GCA_002746175.1
Gemmatimonadetes bacterium, GB_GCA_002709665.1
Ignavibacteria bacterium UBA6906, GB_GCA_002451155.1
Thermoleophilia bacterium UBA6103, GB_GCA_002423545.1
Candidatus Lambdaproteobacteria bacterium, GB_GCA_002753255.1
Bdellovibrionales bacterium GWA1_52_35, GB_GCA_001769805.1
Actinobacteria bacterium UBA912, GB_GCA_002293965.1
o__SM23-28-2
Armatimonadetes bacterium 13_1_40CM_64_14, GB_GCA_001917855.1
f__Methanosarcinaceae
ANME-2A_00242
ASW-3_02245
ANME-2c-GOS_01327
Methanosarcinales archaeon UBA261, GB_GCA_002506015.1
o__Methanosarcinales
o__Methanotrichales_A; f__Methermicoccaceae; g__Methermicoccus
o__Methanotrichales; f__Methanotrichaceae
0.10

B.

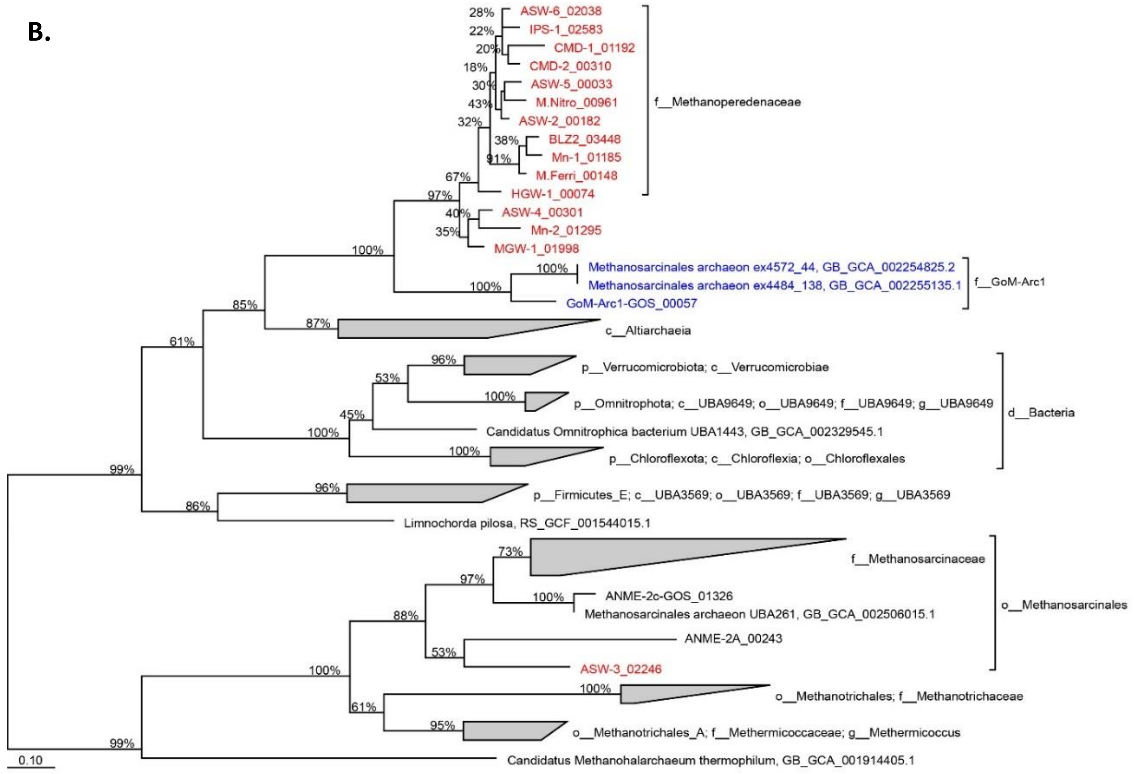

ASW-6_02038
IPS-1_02583
CMD-1_01192
CMD-2_00310
ASW-5_00033
M.Nitro_00961
ASW-2_00182
BLZ2_03448
Mn-1_01185
M.Ferri_00148
HGW-1_00074
ASW-4_00301
Mn-2_01295
MGW-1_01998
f__Methanoperedenaceae
Methanosarcinales archaeon ex4572_44, GB_GCA_002254825.2
Methanosarcinales archaeon ex4484_138, GB_GCA_002255135.1
GoM-Arc1-GOS_00057
f__GoM-Arc1
c__Altiarchaeia
p__Verrucomicrobiota; c__Verrucomicrobiae
p__Omnitrophota; c__UBA9649; o__UBA9649; f__UBA9649; g__UBA9649
Candidatus Omnitrophica bacterium UBA1443, GB_GCA_002329545.1
p__Chloroflexota; c__Chloroflexia; o__Chloroflexales
d__Bacteria
p__Firmicutes_E; c__UBA3569; o__UBA3569; f__UBA3569; g__UBA3569
Limnochorda pilosa, RS_GCF_001544015.1
f__Methanosarcinaceae
ANME-2c-GOS_01326
Methanosarcinales archaeon UBA261, GB_GCA_002506015.1
ANME-2A_00243
ASW-3_02246
o__Methanosarcinales
o__Methanotrichales; f__Methanotrichaceae
o__Methanotrichales_A; f__Methermicoccaceae; g__Methermicoccus
Candidatus Methanohalarchaeum thermophilum, GB_GCA_001914405.1
0.10

C.

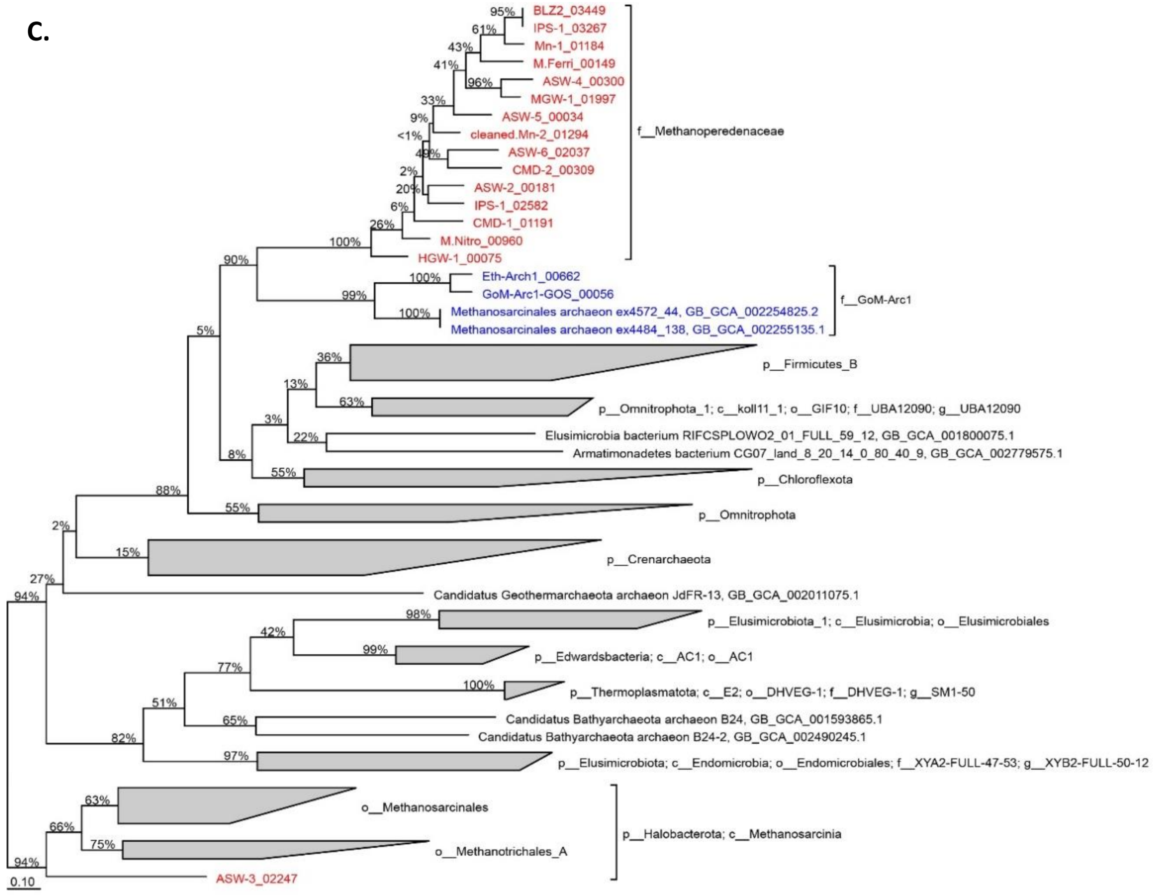

BLZ2_03449
IPS-1_03267
Mn-1_01184
M.Ferri_00149
ASW-4_00300
MGW-1_01997
ASW-5_00034
cleaned.Mn-2_01294
ASW-6_02037
CMD-2_00309
ASW-2_00181
IPS-1_02582
CMD-1_01191
M.Nitro_00960
HGW-1_00075
f__Methanoperedenaceae
Eth-Arch1_00662
GoM-Arc1-GOS_00056
Methanosarcinales archaeon ex4572_44, GB_GCA_002254825.2
Methanosarcinales archaeon ex4484_138, GB_GCA_002255135.1
f__GoM-Arc1
p__Firmicutes_B
p__Omnitrophota_1; c__koll11_1; o__GIF10; f__UBA12090; g__UBA12090
Elusimicrobia bacterium RIFCSPLOWO2_01_FULL_59_12, GB_GCA_001800075.1
Armatimonadetes bacterium CG07_land_8_20_14_0_80_40_9, GB_GCA_002779575.1
p__Chloroflexota
p__Omnitrophota
p__Crenarchaeota
Candidatus Geothermarchaeota archaeon JdFR-13, GB_GCA_002011075.1
p__Elusimicrobiota_1; c__Elusimicrobia; o__Elusimicrobiales
p__Edwardsbacteria; c__AC1; o__AC1
p__Thermoplasmatota; c__E2; o__DHVEG-1; f__DHVEG-1; g__SM1-50
Candidatus Bathyarchaeota archaeon B24, GB_GCA_001593865.1
Candidatus Bathyarchaeota archaeon B24-2, GB_GCA_002490245.1
p__Elusimicrobiota; c__Endomicrobia; o__Endomicrobiales; f__XYA2-FULL-47-53; g__XYB2-FULL-50-12
o__Methanosarcinales
o__Methanotrichales_A
p__Halobacterota; c__Methanosarcinia
ASW-3_02247
95%
61%
43%
41%
96%
33%
9%
<1%
49%
2%
20%
6%
26%
100%
90%
100%
99%
100%
5%
36%
13%
63%
3%
22%
8%
55%
88%
55%
2%
15%
27%
94%
98%
42%
99%
77%
100%
51%
65%
82%
97%
63%
66%
75%
94%
0.10

D.

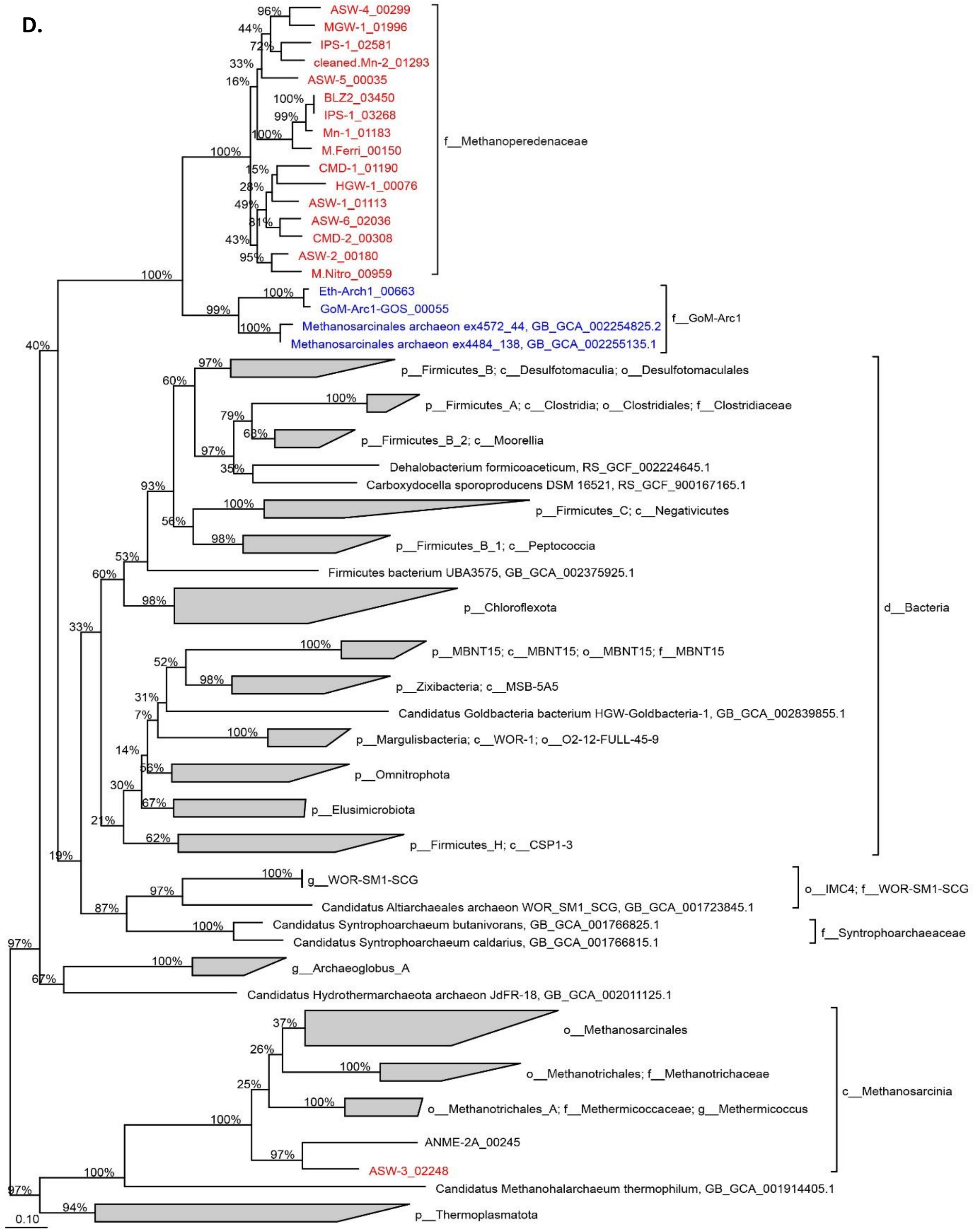
ASW-4_00299
MGW-1_01996
IPS-1_02581
cleaned.Mn-2_01293
ASW-5_00035
BLZ2_03450
IPS-1_03268
Mn-1_01183
M.Ferri_00150
CMD-1_01190
HGW-1_00076
ASW-1_01113
ASW-6_02036
CMD-2_00308
ASW-2_00180
M.Nitro_00959
f__Methanoperedenaceae
Eth-Arch1_00663
GoM-Arc1-GOS_00055
Methanosarcinales archaeon ex4572_44, GB_GCA_002254825.2
Methanosarcinales archaeon ex4484_138, GB_GCA_002255135.1
f__GoM-Arc1
p__Firmicutes_B; c__Desulfotomaculia; o__Desulfotomaculales
p__Firmicutes_A; c__Clostridia; o__Clostridiales; f__Clostridiaceae
p__Firmicutes_B_2; c__Moorellia
Dehalobacterium formicoaceticum, RS_GCF_002224645.1
Carboxydocella sporoproducens DSM 16521, RS_GCF_900167165.1
p__Firmicutes_C; c__Negativicutes
p__Firmicutes_B_1; c__Peptococcia
Firmicutes bacterium UBA3575, GB_GCA_002375925.1
p__Chloroflexota
p__MBNT15; c__MBNT15; o__MBNT15; f__MBNT15
p__Zixibacteria; c__MSB-5A5
Candidatus Goldbacteria bacterium HGW-Goldbacteria-1, GB_GCA_002839855.1
p__Margulisbacteria; c__WOR-1; o__O2-12-FULL-45-9
p__Omnitrophota
p__Elusimicrobiota
p__Firmicutes_H; c__CSP1-3
d__Bacteria
g__WOR-SM1-SCG
Candidatus Altiarchaeales archaeon WOR_SM1_SCG, GB_GCA_001723845.1
o__IMC4; f__WOR-SM1-SCG
Candidatus Syntrophoarchaeum butanivorans, GB_GCA_001766825.1
Candidatus Syntrophoarchaeum caldarius, GB_GCA_001766815.1
f__Syntrophoarchaeaceae
g__Archaeoglobus_A
Candidatus Hydrothermarchaeota archaeon JdFR-18, GB_GCA_002011125.1
o__Methanosarcinales
o__Methanotrichales; f__Methanotrichaceae
o__Methanotrichales_A; f__Methermicoccaceae; g__Methermicoccus
ANME-2A_00245
ASW-3_02248
c__Methanosarcinia
Candidatus Methanohalarchaeum thermophilum, GB_GCA_001914405.1
p__Thermoplasmatota
0.10

E.

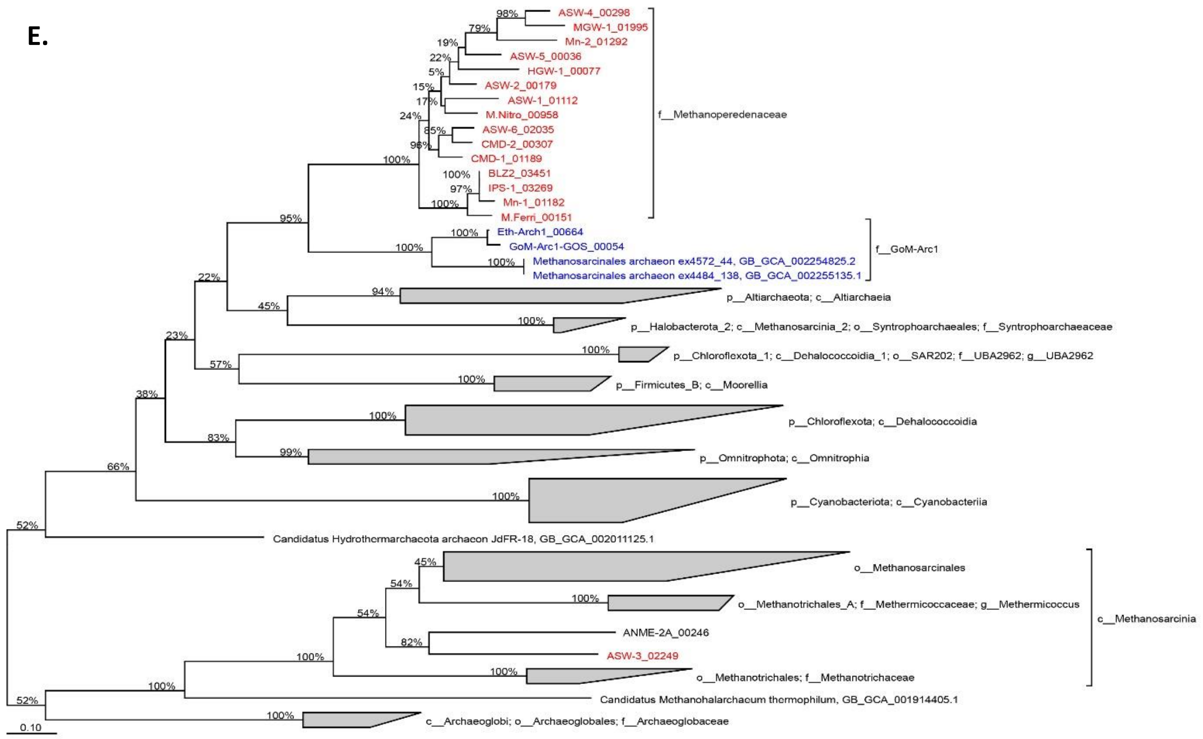
ASW-4_00298
MGW-1_01995
Mn-2_01292
ASW-5_00036
HGW-1_00077
ASW-2_00179
ASW-1_01112
M.Nitro_00958
ASW-6_02035
CMD-2_00307
CMD-1_01189
BLZ2_03451
IPS-1_03269
Mn-1_01182
M.Ferri_00151
f__Methanoperedenaceae
Eth-Arch1_00664
GoM-Arc1-GOS_00054
Methanosarcinales archaeon ex4572_44, GB_GCA_002254825.2
Methanosarcinales archaeon ex4484_138, GB_GCA_002255135.1
f__GoM-Arc1
p__Altiarchaeota; c__Altiarchaeia
p__Halobacterota_2; c__Methanosarcinia_2; o__Syntrophoarchaeales; f__Syntrophoarchaeaceae
p__Chloroflexota_1; c__Dehalococcoidia_1; o__SAR202; f__UBA2962; g__UBA2962
p__Firmicutes_B; c__Moorellia
p__Chloroflexota; c__Dehalococcoidia
p__Omnitrophota; c__Omnitrophia
p__Cyanobacteriota; c__Cyanobacteriia
Candidatus Hydrothermarchaeota archaeon JdFR-18, GB_GCA_002011125.1
o__Methanosarcinales
o__Methanotrichales_A; f__Methermicoccaceae; g__Methermicoccus
ANME-2A_00246
ASW-3_02249
o__Methanotrichales; f__Methanotrichaceae
c__Methanosarcinia
Candidatus Methanohalarchaeum thermophilum, GB_GCA_001914405.1
c__Archaeoglobi; o__Archaeoglobales; f__Archaeoglobaceae
0.10

F.

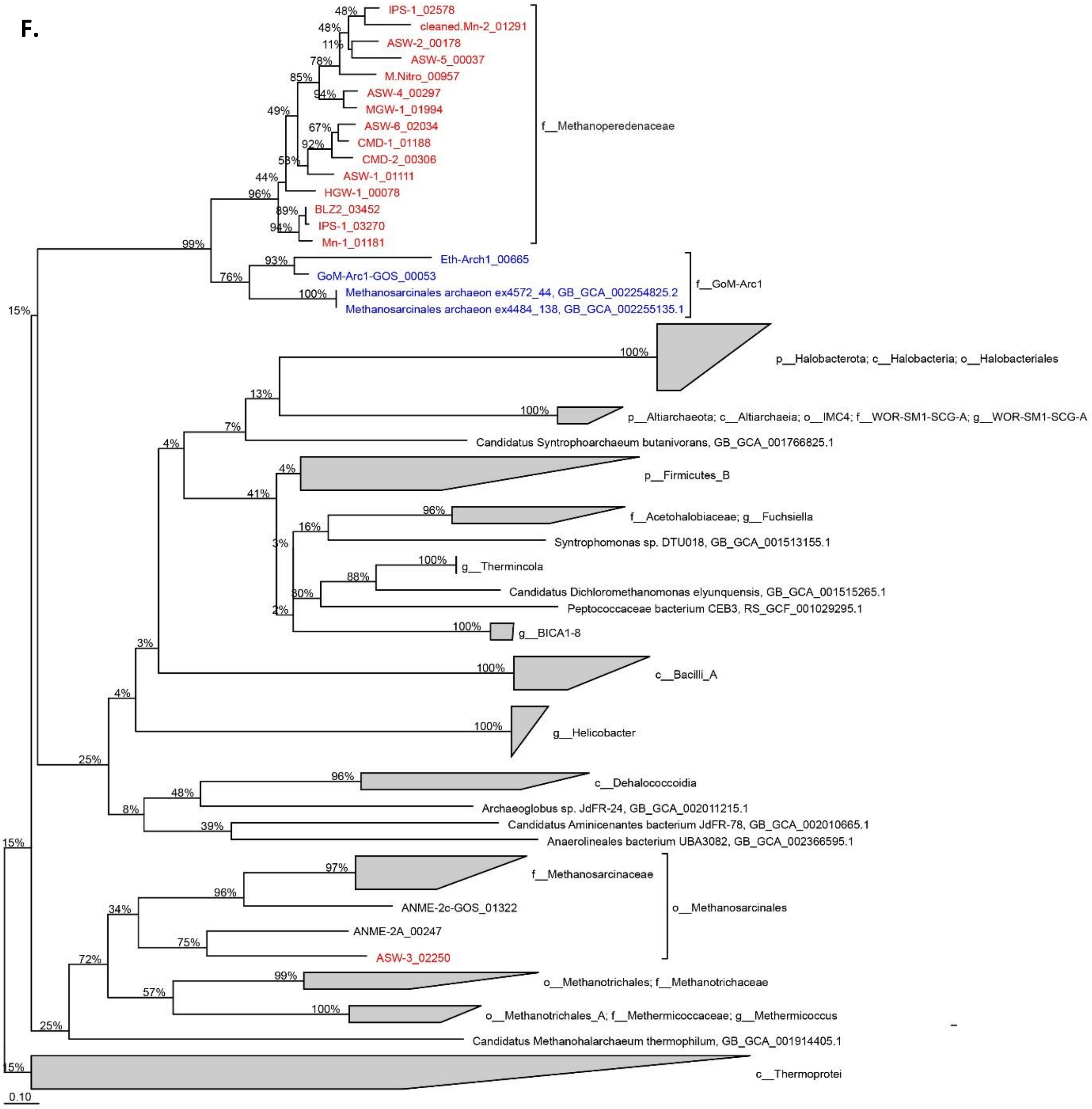
IPS-1_02578
cleaned.Mn-2_01291
ASW-2_00178
ASW-5_00037
M.Nitro_00957
ASW-4_00297
MGW-1_01994
ASW-6_02034
CMD-1_01188
CMD-2_00306
ASW-1_01111
HGW-1_00078
BLZ2_03452
IPS-1_03270
Mn-1_01181
f__Methanoperedenaceae
Eth-Arch1_00665
GoM-Arc1-GOS_00053
Methanosarcinales archaeon ex4572_44, GB_GCA_002254825.2
Methanosarcinales archaeon ex4484_138, GB_GCA_002255135.1
f__GoM-Arc1
p__Halobacterota; c__Halobacteria; o__Halobacteriales
p__Altiarchaeota; c__Altiarchaeia; o__IMC4; f__WOR-SM1-SCG-A; g__WOR-SM1-SCG-A
Candidatus Syntrophoarchaeum butanivorans, GB_GCA_001766825.1
p__Firmicutes_B
f__Acetohalobiaceae; g__Fuchsiella
Syntrophomonas sp. DTU018, GB_GCA_001513155.1
g__Thermincola
Candidatus Dichloromethanomonas elyunquensis, GB_GCA_001515265.1
Peptococcaceae bacterium CEB3, RS_GCF_001029295.1
g__BICA1-8
c__Bacilli_A
g__Helicobacter
c__Dehalococcoidia
Archaeoglobus sp. JdFR-24, GB_GCA_002011215.1
Candidatus Aminicenantes bacterium JdFR-78, GB_GCA_002010665.1
Anaerolineales bacterium UBA3082, GB_GCA_002366595.1
f__Methanosarcinaceae
ANME-2c-GOS_01322
ANME-2A_00247
ASW-3_02250
o__Methanosarcinales
o__Methanotrichales; f__Methanotrichaceae
o__Methanotrichales_A; f__Methermicoccaceae; g__Methermicoccus
Candidatus Methanohalarchaeum thermophilum, GB_GCA_001914405.1
c__Thermoprotei
0.10

G.

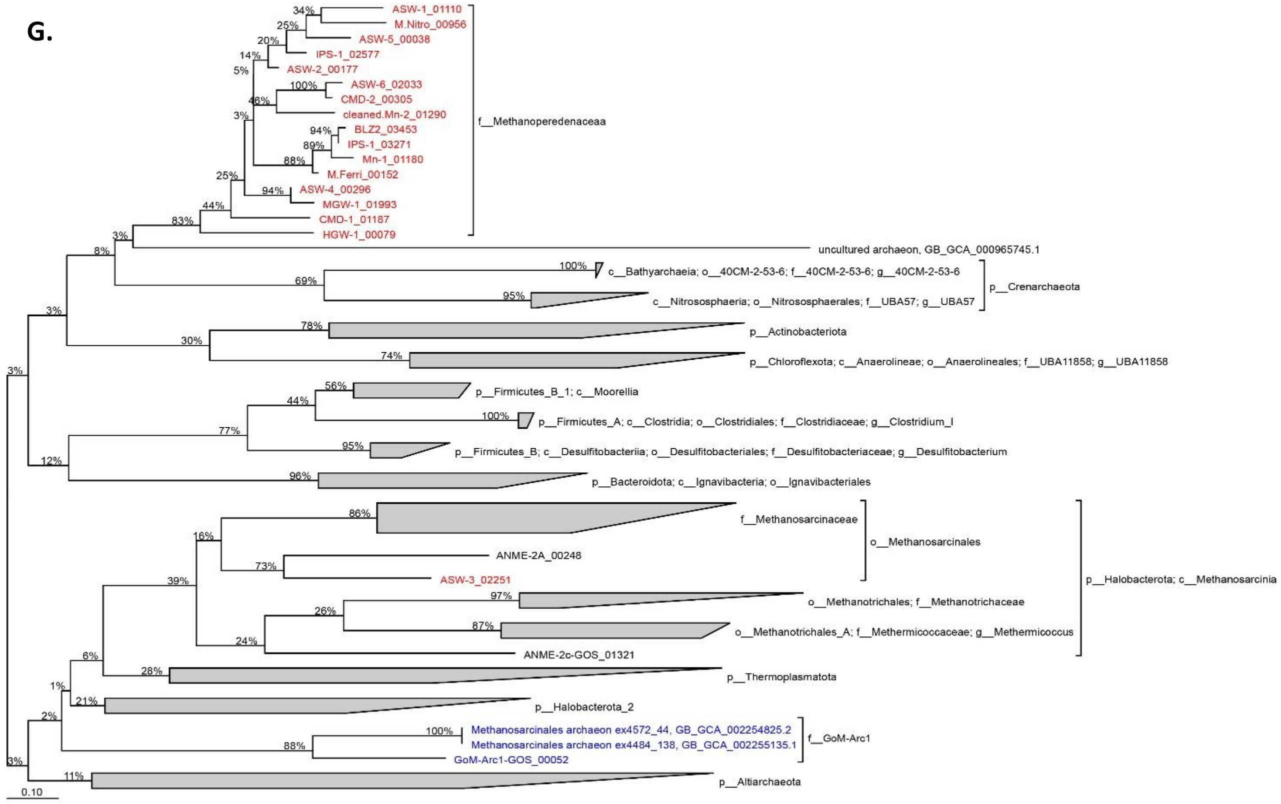
ASW-1_01110
M.Nitro_00956
ASW-5_00038
IPS-1_02577
ASW-2_00177
ASW-6_02033
CMD-2_00305
cleaned.Mn-2_01290
BLZ2_03453
IPS-1_03271
Mn-1_01180
M.Ferri_00152
ASW-4_00296
MGW-1_01993
CMD-1_01187
HGW-1_00079
f__Methanoperedenaceaa
uncultured archaeon, GB_GCA_000965745.1
c__Bathyarchaeia; o__40CM-2-53-6; f__40CM-2-53-6; g__40CM-2-53-6
c__Nitrososphaeria; o__Nitrososphaerales; f__UBA57; g__UBA57
p__Crenarchaeota
p__Actinobacteriota
p__Chloroflexota; c__Anaerolineae; o__Anaerolineales; f__UBA11858; g__UBA11858
p__Firmicutes_B_1; c__Moorellia
p__Firmicutes_A; c__Clostridia; o__Clostridiales; f__Clostridiaceae; g__Clostridium_I
p__Firmicutes_B; c__Desulfitobacteriia; o__Desulfitobacteriales; f__Desulfitobacteriaceae; g__Desulfitobacterium
p__Bacteroidota; c__Ignavibacteria; o__Ignavibacteriales
f__Methanosarcinaceae
o__Methanosarcinales
ANME-2A_00248
ASW-3_02251
p__Halobacterota; c__Methanosarcinia
o__Methanotrichales; f__Methanotrichaceae
o__Methanotrichales_A; f__Methermicoccaceae; g__Methermicoccus
ANME-2c-GOS_01321
p__Thermoplasmatota
p__Halobacterota_2
Methanosarcinales archaeon ex4572_44, GB_GCA_002254825.2
Methanosarcinales archaeon ex4484_138, GB_GCA_002255135.1
GoM-Arc1-GOS_00052
f__GoM-Arc1
p__Altiarchaeota
34%
25%
20%
14%
5%
100%
46%
3%
94%
89%
88%
25%
94%
44%
83%
3%
8%
100%
69%
95%
3%
78%
30%
74%
3%
56%
44%
100%
77%
95%
12%
96%
86%
16%
73%
39%
97%
26%
87%
24%
6%
28%
1%
21%
2%
100%
88%
3%
11%
0.10

H.

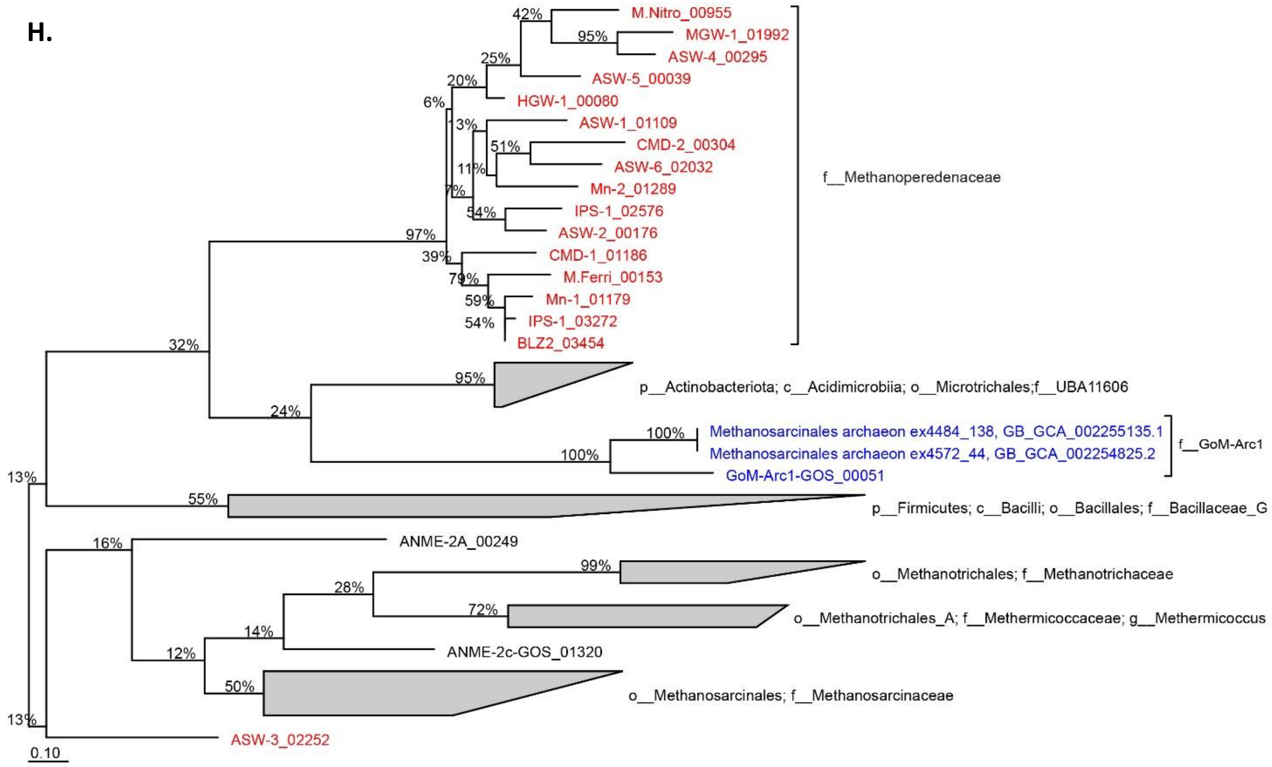
M.Nitro_00955
MGW-1_01992
ASW-4_00295
ASW-5_00039
HGW-1_00080
ASW-1_01109
CMD-2_00304
ASW-6_02032
Mn-2_01289
IPS-1_02576
ASW-2_00176
CMD-1_01186
M.Ferri_00153
Mn-1_01179
IPS-1_03272
BLZ2_03454
f__Methanoperedenaceae
p__Actinobacteriota; c__Acidimicrobiia; o__Microtrichales;f__UBA11606
Methanosarcinales archaeon ex4484_138, GB_GCA_002255135.1
Methanosarcinales archaeon ex4572_44, GB_GCA_002254825.2
GoM-Arc1-GOS_00051
f__GoM-Arc1
p__Firmicutes; c__Bacilli; o__Bacillales; f__Bacillaceae_G
ANME-2A_00249
o__Methanotrichales; f__Methanotrichaceae
o__Methanotrichales_A; f__Methermicoccaceae; g__Methermicoccus
ANME-2c-GOS_01320
o__Methanosarcinales; f__Methanosarcinaceae
ASW-3_02252
42%
95%
25%
20%
6%
3%
51%
11%
7%
54%
97%
39%
79%
59%
54%
32%
95%
24%
100%
100%
13%
55%
16%
99%
28%
72%
14%
12%
50%
13%
0.10

I.

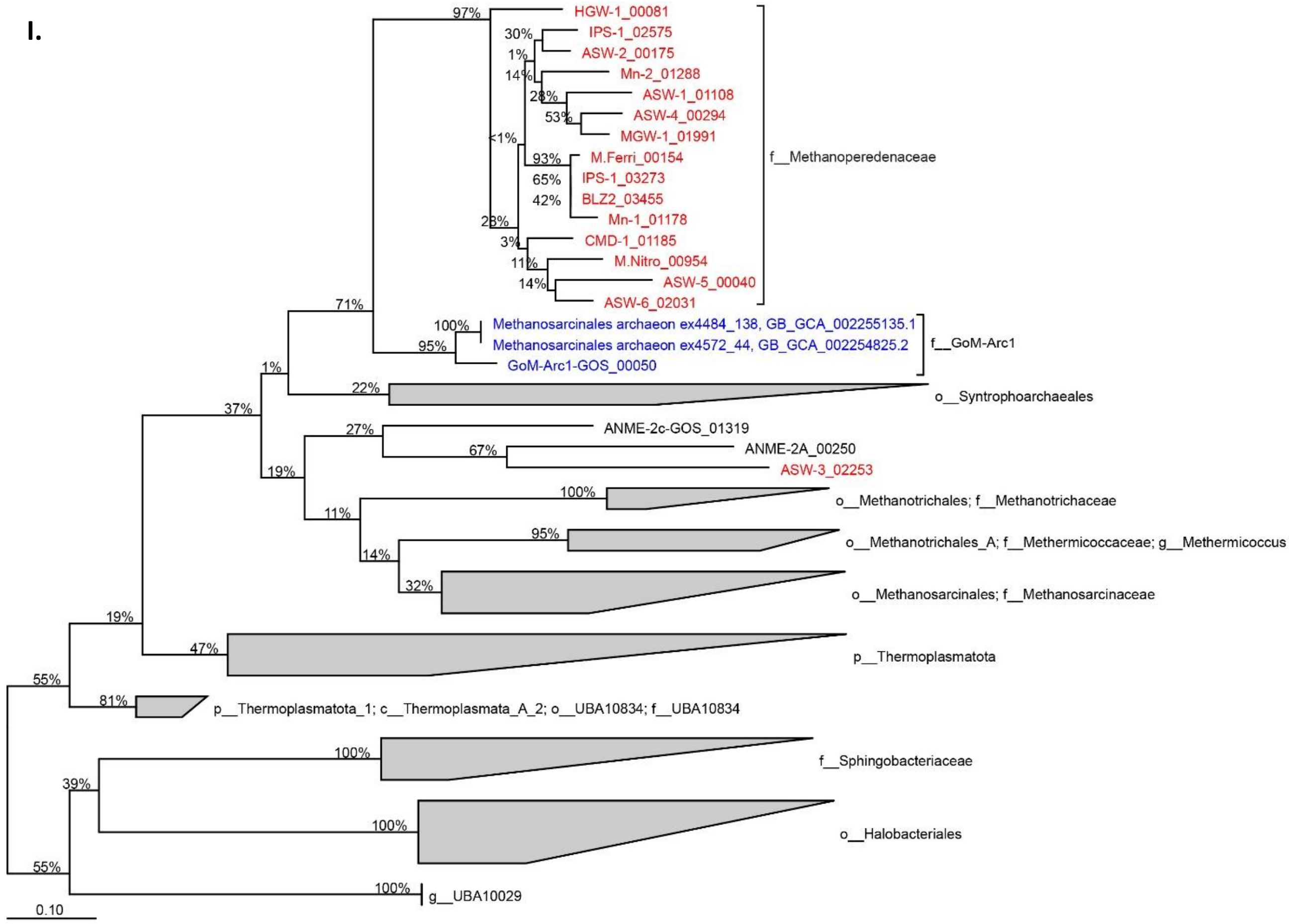
HGW-1_00081
IPS-1_02575
ASW-2_00175
Mn-2_01288
ASW-1_01108
ASW-4_00294
MGW-1_01991
M.Ferri_00154
IPS-1_03273
BLZ2_03455
Mn-1_01178
CMD-1_01185
M.Nitro_00954
ASW-5_00040
ASW-6_02031
f__Methanoperedenaceae
Methanosarcinales archaeon ex4484_138, GB_GCA_002255135.1
Methanosarcinales archaeon ex4572_44, GB_GCA_002254825.2
GoM-Arc1-GOS_00050
f__GoM-Arc1
o__Syntrophoarchaeales
ANME-2c-GOS_01319
ANME-2A_00250
ASW-3_02253
o__Methanotrichales; f__Methanotrichaceae
o__Methanotrichales_A; f__Methermicoccaceae; g__Methermicoccus
o__Methanosarcinales; f__Methanosarcinaceae
p__Thermoplasmatota
p__Thermoplasmatota_1; c__Thermoplasmata_A_2; o__UBA10834; f__UBA10834
f__Sphingobacteriaceae
o__Halobacteriales
g__UBA10029
0.10

J.

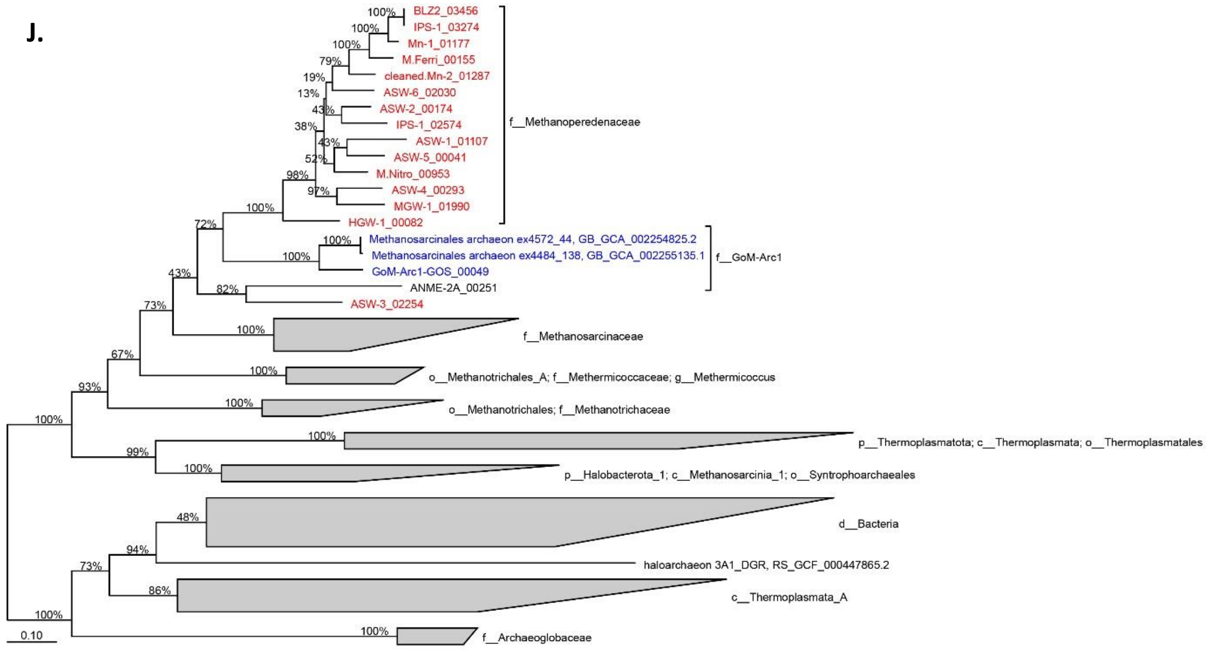
BLZ2_03456
IPS-1_03274
Mn-1_01177
M.Ferri_00155
cleaned.Mn-2_01287
ASW-6_02030
ASW-2_00174
IPS-1_02574
ASW-1_01107
ASW-5_00041
M.Nitro_00953
ASW-4_00293
MGW-1_01990
HGW-1_00082
f__Methanoperedenaceae
Methanosarcinales archaeon ex4572_44, GB_GCA_002254825.2
Methanosarcinales archaeon ex4484_138, GB_GCA_002255135.1
GoM-Arc1-GOS_00049
f__GoM-Arc1
ANME-2A_00251
ASW-3_02254
f__Methanosarcinaceae
o__Methanotrichales_A; f__Methermicoccaceae; g__Methermicoccus
o__Methanotrichales; f__Methanotrichaceae
p__Thermoplasmatota; c__Thermoplasmata; o__Thermoplasmatales
p__Halobacterota_1; c__Methanosarcinia_1; o__Syntrophoarchaeales
d__Bacteria
haloarchaeon 3A1_DGR, RS_GCF_000447865.2
c__Thermoplasmata_A
f__Archaeoglobaceae
0.10

K.

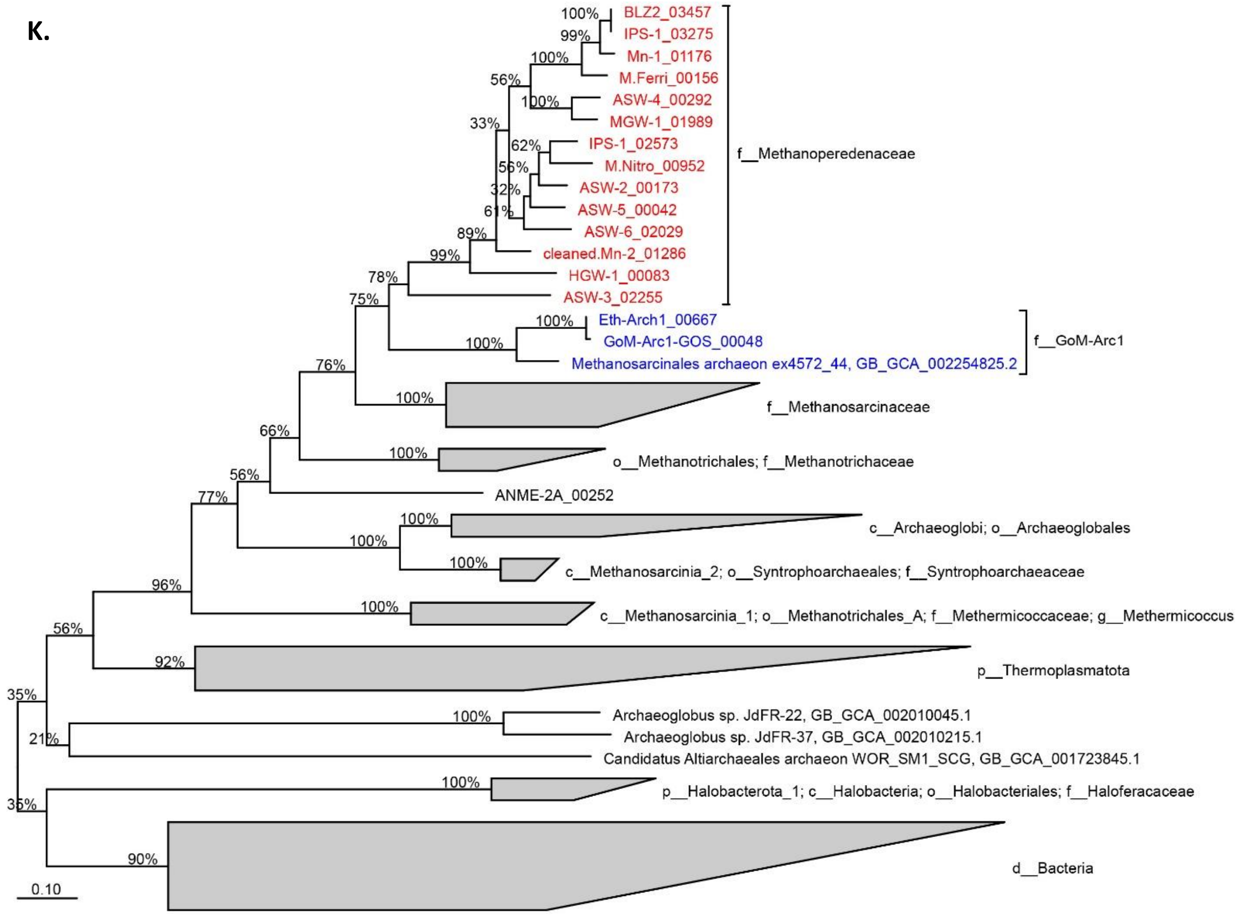

BLZ2_03457
IPS-1_03275
Mn-1_01176
M.Ferri_00156
ASW-4_00292
MGW-1_01989
IPS-1_02573
M.Nitro_00952
ASW-2_00173
ASW-5_00042
ASW-6_02029
cleaned.Mn-2_01286
HGW-1_00083
ASW-3_02255
f__Methanoperedenaceae
Eth-Arch1_00667
GoM-Arc1-GOS_00048
Methanosarcinales archaeon ex4572_44, GB_GCA_002254825.2
f__GoM-Arc1
f__Methanosarcinaceae
o__Methanotrichales; f__Methanotrichaceae
ANME-2A_00252
c__Archaeoglobi; o__Archaeoglobales
c__Methanosarcinia_2; o__Syntrophoarchaeales; f__Syntrophoarchaeaceae
c__Methanosarcinia_1; o__Methanotrichales_A; f__Methermicoccaceae; g__Methermicoccus
p__Thermoplasmatota
Archaeoglobus sp. JdFR-22, GB_GCA_002010045.1
Archaeoglobus sp. JdFR-37, GB_GCA_002010215.1
Candidatus Altiarchaeales archaeon WOR_SM1_SCG, GB_GCA_001723845.1
p__Halobacterota_1; c__Halobacteria; o__Halobacteriales; f__Haloferacaceae
d__Bacteria
0.10

L.

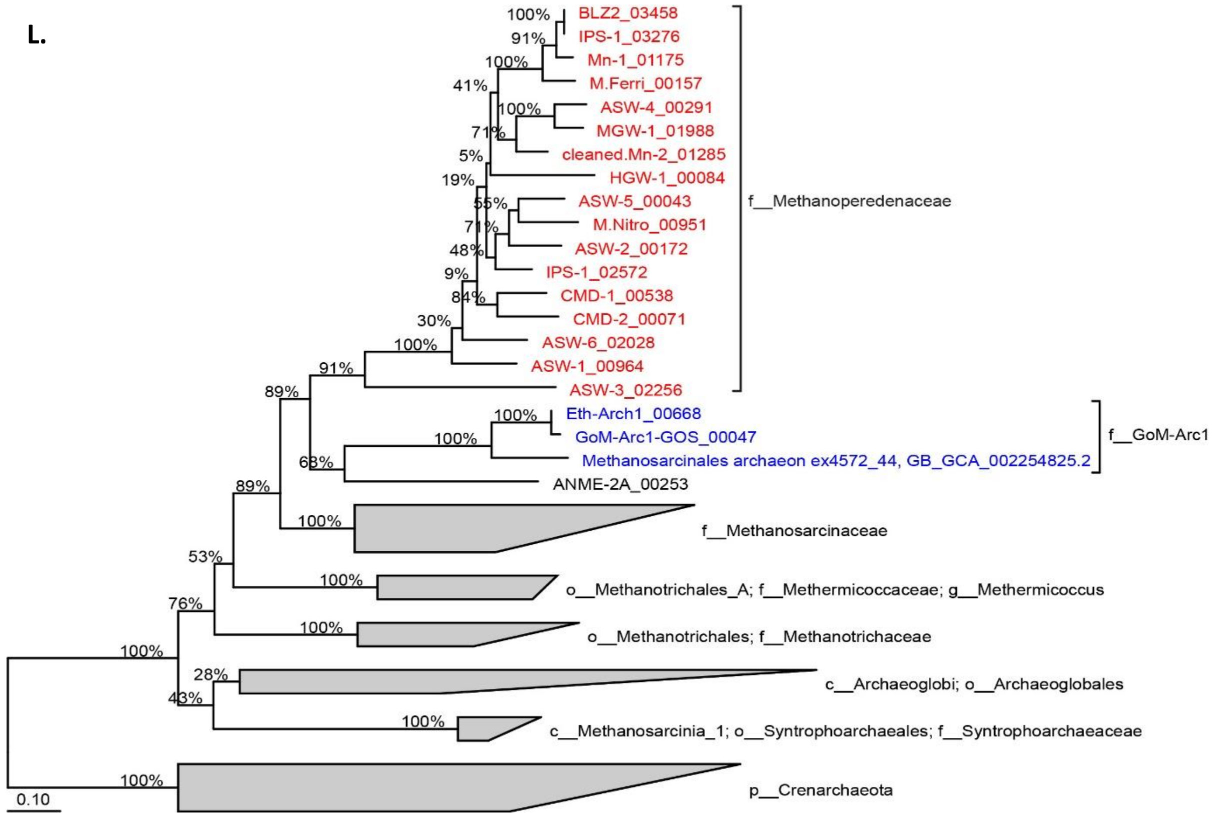

BLZ2_03458
IPS-1_03276
Mn-1_01175
M.Ferri_00157
ASW-4_00291
MGW-1_01988
cleaned.Mn-2_01285
HGW-1_00084
ASW-5_00043
M.Nitro_00951
ASW-2_00172
IPS-1_02572
CMD-1_00538
CMD-2_00071
ASW-6_02028
ASW-1_00964
ASW-3_02256
f__Methanoperedenaceae
Eth-Arch1_00668
GoM-Arc1-GOS_00047
Methanosarcinales archaeon ex4572_44, GB_GCA_002254825.2
f__GoM-Arc1
ANME-2A_00253
f__Methanosarcinaceae
o__Methanotrichales_A; f__Methermicoccaceae; g__Methermicoccus
o__Methanotrichales; f__Methanotrichaceae
c__Archaeoglobi; o__Archaeoglobales
c__Methanosarcinia_1; o__Syntrophoarchaeales; f__Syntrophoarchaeaceae
p__Crenarchaeota
0.10

M.

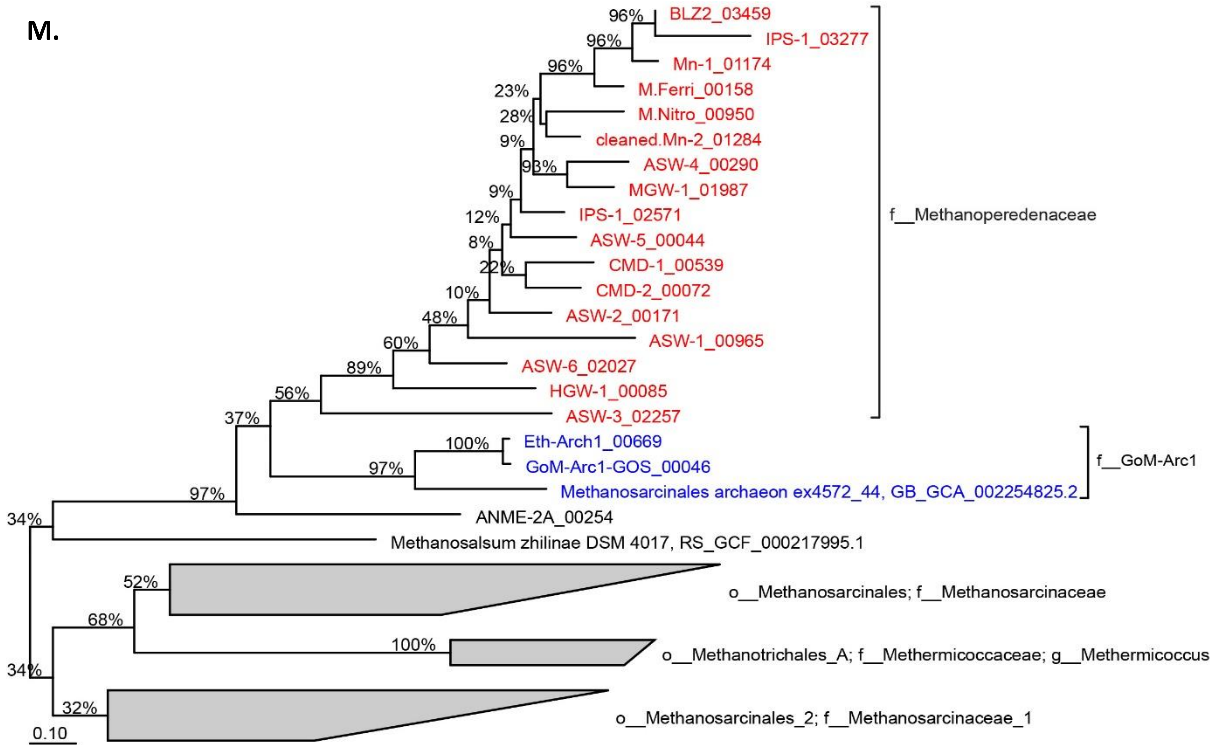

Figure S6. Phylogenetic analysis of the Fpo subunits annotated in the *Methanoperedenaceae* genomes. A. FpoA **B.** FpoB **C.** FpoC **D.** FpoD **E.** FpoH **F.** FpoI **G.** $FpoJ_1$ **H.** $FpoJ_2$ **I.** FpoK **J.** FpoL **K.** FpoM **L.** FpoN **M.** FpoO. Putative genes recovered from the *Methanoperedenaceae* are highlighted in red. The gene trees were inferred using maximum likelihood and support values calculated via non-parametric bootstrapping. Reference genes and the taxonomy are from the GTDB v83 database (D.H. Parks, M. Chuvochina, D. W. Waite, C. Rinke, A. Skarshewski, P.-A. Chaumeil and P. Hugenholtz, Nat Biotechnol 36: 996-1004, 2018, https://doi.org/10.1038/nbt.4229).
