# Supplementary material for: Lateral Gene Transfer Drives Metabolic Flexibility in the Anaerobic Methane-Oxidizing Archaeal Family Methanoperedenaceae
Source: mBio. 2020 Jun 30;11(3):e01325-20. doi: 10.1128/mBio.01325-20 (PMC7327174; doi:10.1128/mBio.01325-20)
Supplement: FIG S7 [file mBio.01325-20-sf007.pdf]

A.

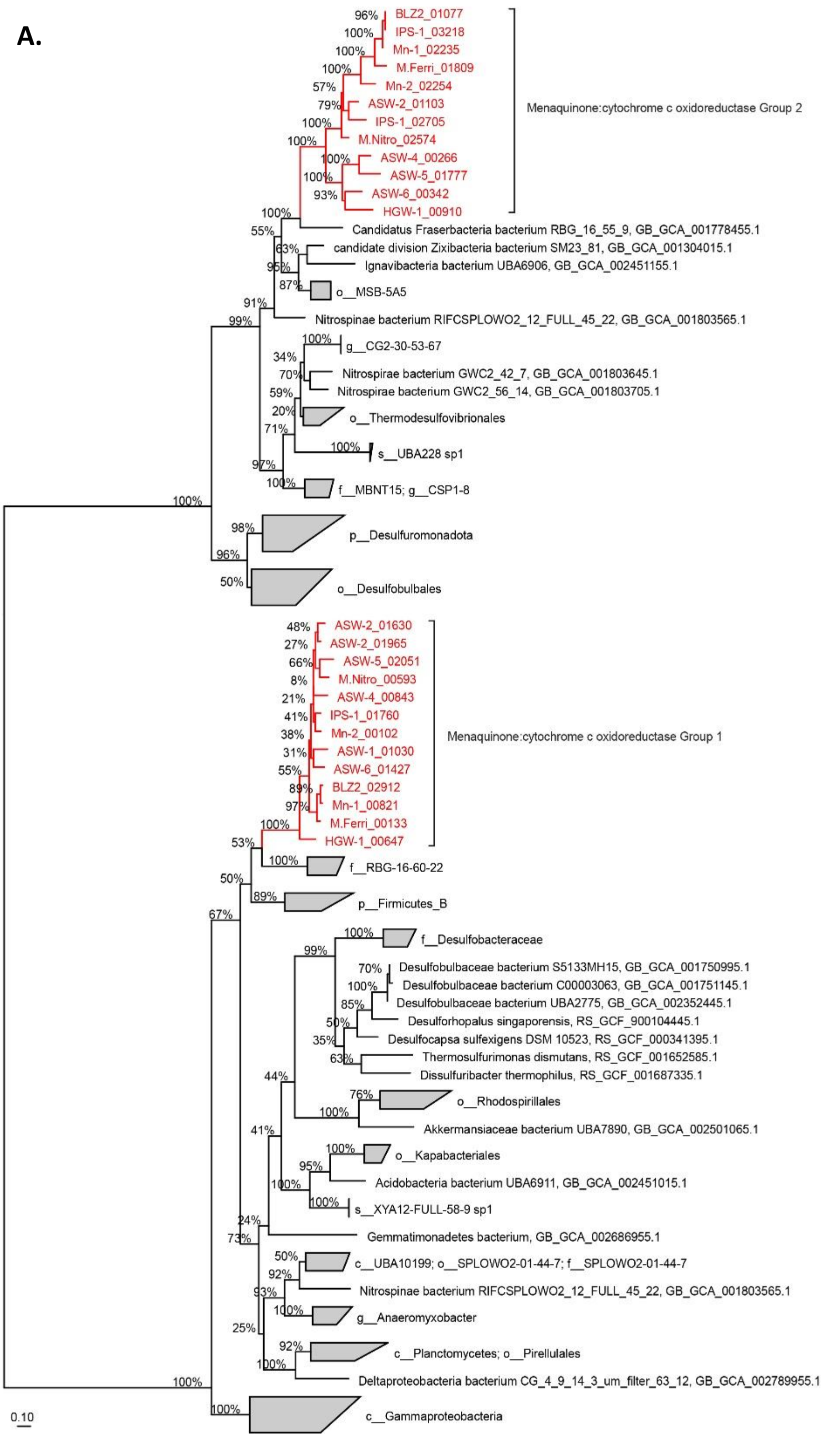

BLZ2_01077
IPS-1_03218
Mn-1_02235
M.Ferri_01809
Mn-2_02254
ASW-2_01103
IPS-1_02705
M.Nitro_02574
ASW-4_00266
ASW-5_01777
ASW-6_00342
HGW-1_00910
Menaquinone:cytochrome c oxidoreductase Group 2
Candidatus Fraserbacteria bacterium RBG_16_55_9, GB_GCA_001778455.1
candidate division Zixibacteria bacterium SM23_81, GB_GCA_001304015.1
Ignavibacteria bacterium UBA6906, GB_GCA_002451155.1
o__MSB-5A5
Nitrospinae bacterium RIFCSPLOWO2_12_FULL_45_22, GB_GCA_001803565.1
g__CG2-30-53-67
Nitrospirae bacterium GWC2_42_7, GB_GCA_001803645.1
Nitrospirae bacterium GWC2_56_14, GB_GCA_001803705.1
o__Thermodesulfovibrionales
s__UBA228 sp1
f__MBNT15; g__CSP1-8
p__Desulfuromonadota
o__Desulfobulbales
ASW-2_01630
ASW-2_01965
ASW-5_02051
M.Nitro_00593
ASW-4_00843
IPS-1_01760
Mn-2_00102
ASW-1_01030
ASW-6_01427
BLZ2_02912
Mn-1_00821
M.Ferri_00133
HGW-1_00647
Menaquinone:cytochrome c oxidoreductase Group 1
f__RBG-16-60-22
p__Firmicutes_B
f__Desulfobacteraceae
Desulfobulbaceae bacterium S5133MH15, GB_GCA_001750995.1
Desulfobulbaceae bacterium C00003063, GB_GCA_001751145.1
Desulfobulbaceae bacterium UBA2775, GB_GCA_002352445.1
Desulforhopalus singaporensis, RS_GCF_900104445.1
Desulfocapsa sulfexigens DSM 10523, RS_GCF_000341395.1
Thermosulfurimonas dismutans, RS_GCF_001652585.1
Dissulfuribacter thermophilus, RS_GCF_001687335.1
o__Rhodospirillales
Akkermansiaceae bacterium UBA7890, GB_GCA_002501065.1
o__Kapabacteriales
Acidobacteria bacterium UBA6911, GB_GCA_002451015.1
s__XYA12-FULL-58-9 sp1
Gemmatimonadetes bacterium, GB_GCA_002686955.1
c__UBA10199; o__SPLOWO2-01-44-7; f__SPLOWO2-01-44-7
Nitrospinae bacterium RIFCSPLOWO2_12_FULL_45_22, GB_GCA_001803565.1
g__Anaeromyxobacter
c__Planctomycetes; o__Pirellulales
Deltaproteobacteria bacterium CG_4_9_14_3_um_filter_63_12, GB_GCA_002789955.1
c__Gammaproteobacteria
0.10

B.

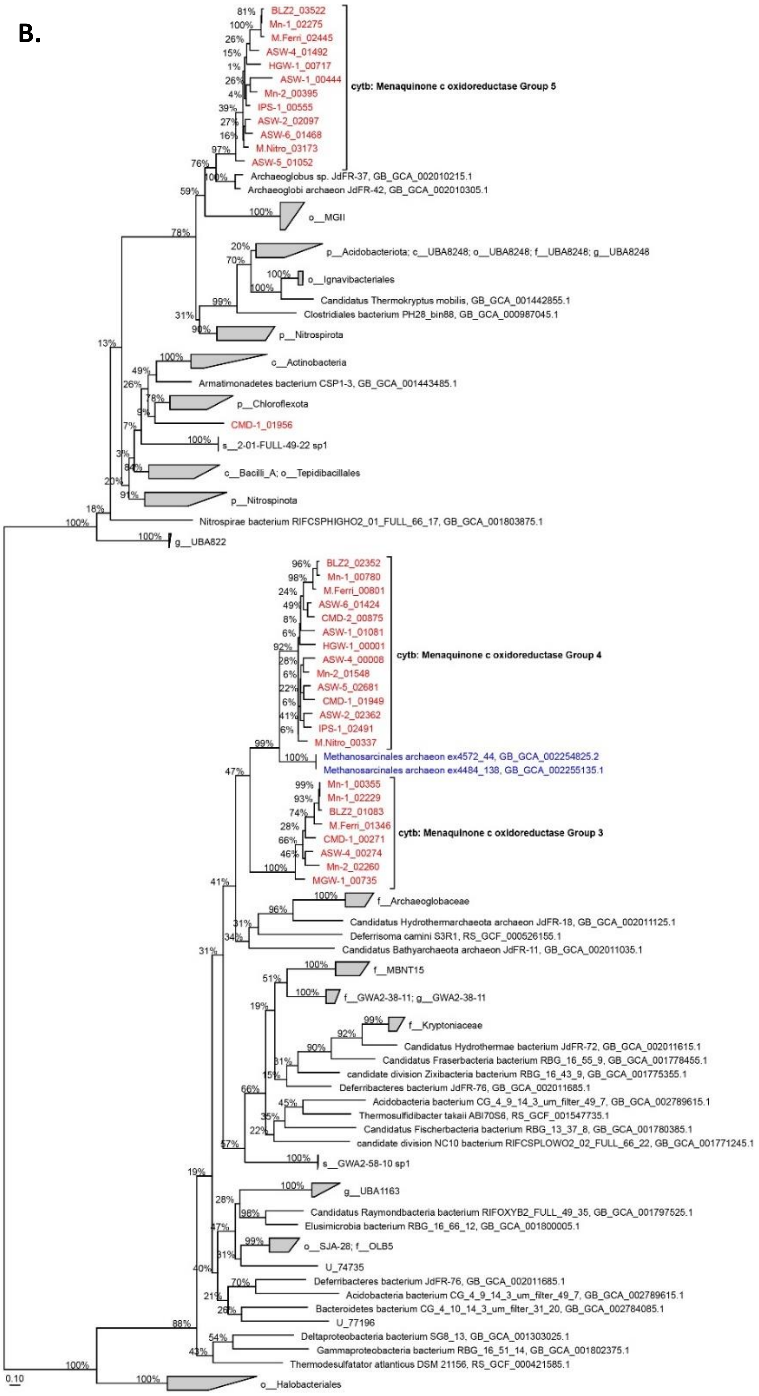

Figure S7. Phylogenetic analysis of the subunits of the MK:cytochrome oxidoreductases annotated in the *Methanoperedenaceae* MAGs. A. Analysis of the NrfD subunits. **B.** Analysis of the b-type cytochromes. Bootstrap values for the maximum-likelihood trees were determined using non-parametric bootstrapping with 100 replicates. The scale bars represents amino acid changes.
