# Supplementary figures and images for: Lateral Gene Transfer Drives Metabolic Flexibility in the Anaerobic Methane-Oxidizing Archaeal Family Methanoperedenaceae
Source: mBio. 2020 Jun 30;11(3):e01325-20. doi: 10.1128/mBio.01325-20 (PMC7327174; doi:10.1128/mBio.01325-20)

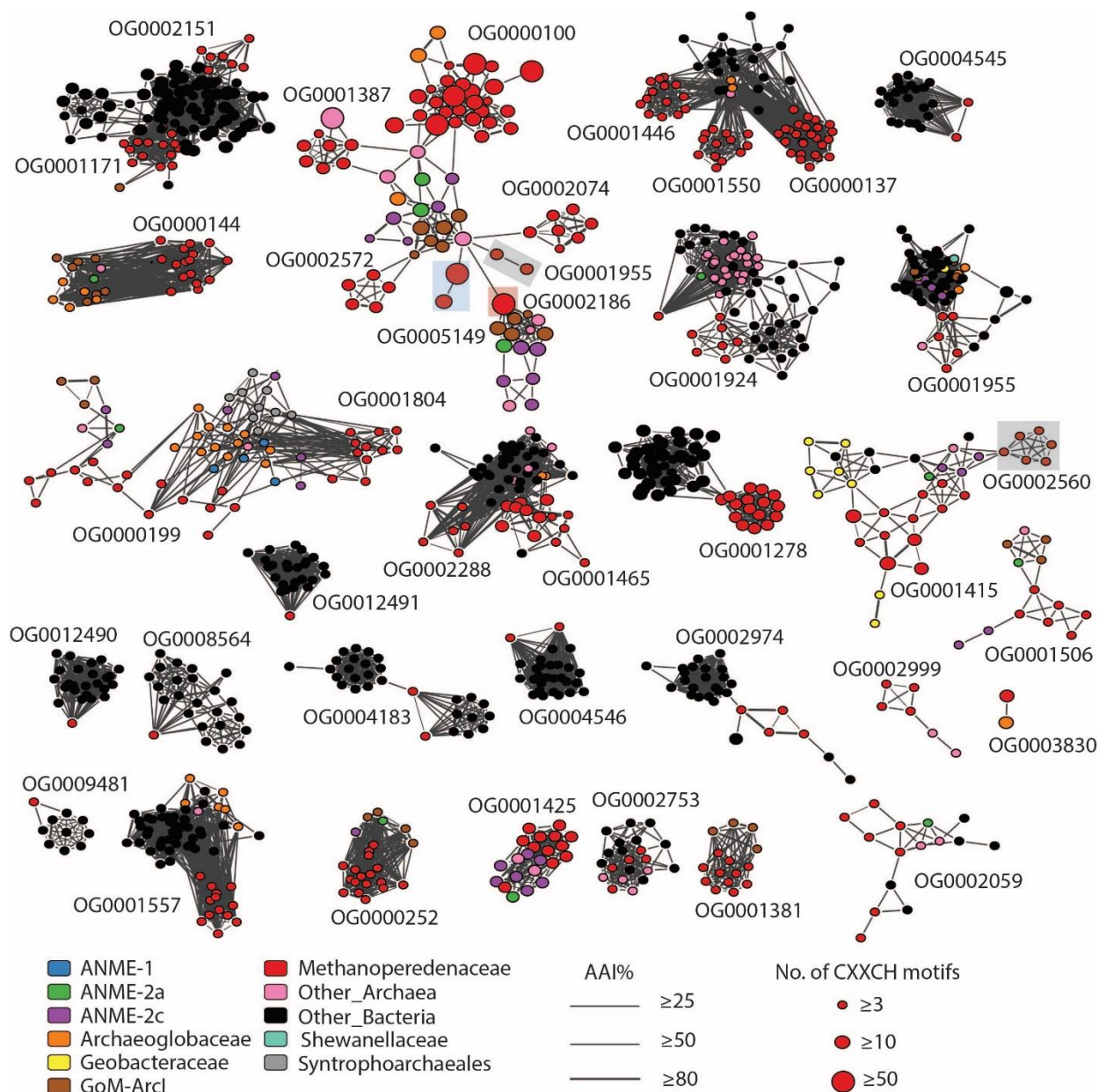

Supplement: FIG S9 [file mBio.01325-20-sf009.pdf]
